# Probiotic *Lactobacillus reuteri* Y7 Protects Against Blue Light–Induced Retinal Degeneration via Antioxidant Defense, Anti-Inflammatory Action, and Gut–Retina Axis Modulation

**DOI:** 10.3390/antiox14121428

**Published:** 2025-11-27

**Authors:** Shu-Ping Tsao, Tu-Hsueh Yeh, Tsung-Jen Wang, Keita Nishiyama, Chun-Hsu Pan, Chih-Hsuan Lou, Hui-Yu Huang

**Affiliations:** 1 Graduate Institute of Metabolism and Obesity Sciences, College of Nutrition, Taipei Medical University, Taipei 11031, Taiwan; d343105004@tmu.edu.tw (S.-P.T.); do2739@tmu.edu.tw (T.-H.Y.); ma48110007@tmu.edu.tw (C.-H.L.); 2 Ph.D. Program in Drug Discovery and Development Industry, College of Pharmacy, Taipei Medical University, Taipei 11031, Taiwan; panch@tmu.edu.tw; 3 Neuroscience Research Center, Taipei Medical University, Taipei 11031, Taiwan; 4 Department of Neurology, Taipei Medical University Hospital, Taipei 11031, Taiwan; 5 Department of Neurology, School of Medicine, College of Medicine, Taipei Medical University, Taipei 11031, Taiwan; 6 International Master/Ph.D. Program in Medicine, College of Medicine, Taipei Medical University, Taipei 11031, Taiwan; 7 Department of Ophthalmology, Taipei Medical University Hospital, Taipei 11031, Taiwan; tjw@tmu.edu.tw; 8 Department of Ophthalmology, School of Medicine, College of Medicine, Taipei Medical University, Taipei 11031, Taiwan; 9 Laboratory of Animal Food Function, Graduate School of Agricultural Science, Tohoku University, Sendai 980-8572, Japan; keita.nishiyama.a6@tohoku.ac.jp; 10 Livestock Immunology Unit, International Education and Research Center for Food and Agricultural Immunology (CFAI), Graduate School of Agricultural Science, Tohoku University, Sendai 980-8572, Japan; 11 Research Center for Digestive Medicine, Taipei Medical University Hospital, Taipei 11031, Taiwan; 12 Nutrition Research Center, Taipei Medical University Hospital, Taipei 11031, Taiwan

**Keywords:** blue light-induced retinal degeneration, *Lactobacillus reuteri* Y7, antioxidant defense, anti-inflammatory activity, gut–retina axis, tight junction integrity

## Abstract

Chronic exposure to short-wavelength blue light induces oxidative stress, inflammation, and apoptosis in retinal tissues, contributing to vision loss. This study investigated the protective effects of *Lactobacillus reuteri* Y7, a human-derived probiotic, against blue light–induced retinal damage in mice. Male C57BL/6 mice were exposed to 400 lux blue light for 35 days and received either low- or high-dose Y7, lutein, or no intervention. Retinal morphology, inflammatory gene expression, gut barrier integrity, and gut microbiota composition were assessed. Low-dose Y7 promoted microbial diversity and enrichment of short-chain fatty acid–producing taxa, while high-dose Y7 favored enrichment of *Akkermansia*, *Parasutterella*, and *Bacteroides*, enhancing mucosal barrier function and metabolic regulation. Both doses attenuated retinal inflammation, preserved retinal layers, and improved gut barrier integrity, with high-dose Y7 matching or exceeding lutein’s protective effects. Mechanistic insights suggest a gut–retina axis whereby microbial metabolites modulate oxidative stress, inflammation, and vascular homeostasis. These findings highlight *L. reuteri* Y7 as a potential non-invasive strategy for retinal degeneration prevention, with efficacy comparable to dietary antioxidants. Future studies should explore long-term safety, metabolite-mediated mechanisms, and comparative efficacy with other antioxidants.

## 1. Introduction

Age-related macular degeneration (AMD) is one of the leading causes of irreversible vision loss in the elderly population worldwide, with dry AMD accounting for approximately 80–90% of cases [1]. Unlike neovascular (wet) AMD, which benefits from anti-VEGF therapies, effective treatment options for dry AMD remain limited [2]. Key pathological drivers include oxidative stress, chronic low-grade inflammation, and epithelial barrier disruption.

Among environmental risk factors, chronic exposure to short-wavelength blue light, which is commonly emitted by digital screens and LED lighting, has been suggested—based on experimental evidence in animal models—to contribute to AMD pathogenesis through phototoxic retinal damage, excessive reactive oxygen species (ROS) generation, and activation of inflammatory cascades [3,4,5]. Recent experimental evidence further demonstrates that prolonged blue light exposure disrupts tight junction proteins in retinal pigment epithelial and corneal epithelial cells, thereby compromising barrier integrity and exacerbating tissue vulnerability [6]. Given the multifactorial etiology of AMD, combination strategies that simultaneously target oxidative stress, inflammation, and barrier dysfunction may provide synergistic protection. Probiotics have emerged as promising non-pharmacological interventions with antioxidant, anti-inflammatory, and barrier-protective properties. Several strains of *Lactobacillus* and *Bifidobacterium* have been reported to attenuate oxidative stress and modulate immune responses in ocular, neurodegenerative, and metabolic disease models [7,8,9]. Plant-derived antioxidants such as lutein and anthocyanins have also been shown to mitigate blue light-induced retinal injury in animal and cellular models [7,10].

The gut–retina axis is a bidirectional communication pathway linking gut microbiota composition to retinal health. It has attracted increasing attention, as alterations in gut microbial communities can influence systemic inflammation and oxidative balance, indirectly affecting ocular tissues [11,12,13]. Recent studies indicate that microbial metabolites such as short-chain fatty acids can modulate retinal microglia activation and photoreceptor survival, highlighting the potential of microbiota-targeted therapies in retinal degeneration [14].

This study aimed to evaluate the protective effects of oral *Lactobacillus reuteri* Y7 in a murine model of chronic blue light exposure that mimics an oxidative stress–driven retinal degeneration paradigm with AMD-like features. To our knowledge, this is the first report directly comparing the efficacy of a single probiotic strain to a plant-derived antioxidant (lutein) in attenuating blue light-induced retinal injury. We hypothesized that Y7 would protect against retinal degeneration through antioxidant defense enhancement, inflammation suppression, and epithelial barrier preservation, potentially via modulation of the gut–retina axis.

## 2. Materials and Methods

### 2.1. Experimental Animals

**Figure 1:**
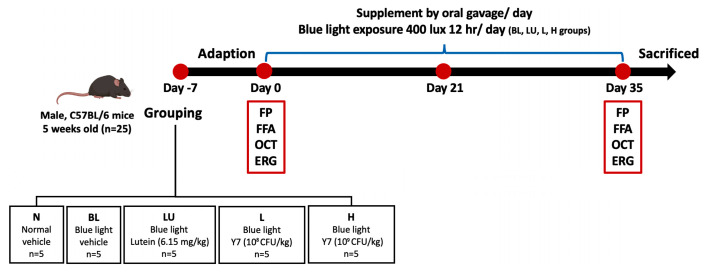
Experimental design and timeline of interventions. Schematic representation of the 35-day animal experiment showing group allocation: NC (normal control), BL (blue light exposure only), LU (blue light + lutein), L (blue light + low-dose *L. reuteri* Y7), and H (blue light + high-dose *L. reuteri* Y7). Blue light exposure protocol, supplementation schedules, and sampling points are indicated.

Five-week-old male C57BL/6 mice (25 g) were obtained from the National Laboratory Animal Center (Taipei, Taiwan) and housed under controlled temperature (22 ± 2 °C), humidity (55 ± 5%), and a 12 h light/dark cycle with ad libitum access to food and water. After one week of acclimation, mice were randomly assigned to five groups (*n* = 5/group): control (NC), blue light only (BL), lutein (LU), and probiotics at low (L) or high (H) doses. The NC group received distilled water by gavage; LU received lutein; L and H received different doses of *Lactobacillus reuteri* Y7. All interventions were administered concurrently with blue light exposure for 35 days (Figure 1). The protocol was approved by the TMU IACUC (LAC-2021-0212).

### 2.2. Preparation of Lactobacillus reuteri Y7

The probiotic *Lactobacillus reuteri* Y7 (10^11^ CFU/g) was provided by Hui-Yu Huang’s lab at Taipei Medical University (Taipei, Taiwan) and originally isolated from healthy breast milk. It was deposited at the Bioresource Collection and Research Center (Taiwan; ID: BCRC 911162). Before oral administration, the Y7 powder was weighed and suspended in distilled water to achieve a final concentration of 10^10^ colony-forming units (CFU)/mL.

### 2.3. Preparation of Lutein

The lutein dosage was determined based on the Taiwan FDA recommendation of 30 mg per day for a 60 kg adult, corresponding to 0.5 mg/kg body weight. To convert the human equivalent dose (HED) to the appropriate mouse equivalent dose (AED), we applied the standard body surface area conversion method [15]. The AED was calculated using the formula: AED (mg/kg) = HED (mg/kg) × Km (human)/Km (mouse) = 0.5 mg/kg × 12.3 = 6.15 mg/kg.

### 2.4. Blue Light–Induced Oxidative-Stress Retinal Degeneration Model Mimicking AMD-like Features

Chronic blue light–induced retinal damage was established using custom-built stainless-steel ventilated chambers (Shineteh Industries, Taipei, Taiwan). Mice were exposed to blue LED light with a peak wavelength of 450 ± 10 nm, corresponding to the spectral range commonly associated with retinal phototoxicity. The illumination intensity was maintained at 400 lux under a 12 h light/12 h dark cycle for 35 consecutive days. This wavelength and exposure paradigm were selected based on previously published blue light–induced retinal degeneration models [4], which have consistently demonstrated both functional and structural damage in the mouse retina. Retinal evaluations, including electroretinography (ERG), fundus photography (FP), fluorescein angiography (FFA), and optical coherence tomography (OCT), were performed on days 0 and 35 to assess retinal function and morphology [16].

### 2.5. Electroretinogram (ERG)

Retinal function was assessed using the Celeris electroretinography (ERG) system (Diagnosys LLC, Lowell, MA, USA) after overnight dark adaptation under dim red light. Mice were anesthetized and maintained on a heating pad (37 ± 1 °C). Pupils were dilated with 1% tropicamide (Alcon, Geneva, Switzerland), and corneal hydration was preserved with 0.3% hypromellose gel (Systane, Alcon, Geneva, Switzerland). The active electrode was positioned on the cornea via a stimulator lens, with reference and ground electrodes placed on the scalp and tail, respectively.

Dark-adapted ERG responses were recorded at four flash intensities: 0.01, 0.1, and 1 cd·s/m^2^ for a- and b-wave responses, and 150 cd·s/m^2^ for c-wave responses. The a-wave amplitude, reflecting photoreceptor (rod and cone) activity, was defined as the negative deflection occurring within 0–20 ms after stimulus onset. The b-wave amplitude, representing bipolar and Müller cell activity, was measured as the vertical distance from the a-wave trough to the b-wave peak, typically occurring within 30–80 ms after stimulus onset. The c-wave, representing retinal pigment epithelium (RPE) activity, was elicited by 150 cd·s/m^2^ flashes and recorded over an 80–10,000 ms time window. Although ERG recordings were obtained at four intensities, group comparisons focused on 1 cd·s/m^2^ for a/b-waves and 150 cd·s/m^2^ for c-waves, which are the most sensitive and stable stimulation levels for detecting functional alterations in blue light-induced retinal degeneration. 

### 2.6. Fundus Photography (FP) and Fundus Fluorescein Angiography (FFA)

After anesthesia, mice were placed on an operating platform. Pupils were dilated with 0.125% atropine eye drops (Sinphar, Taipei, Taiwan) for 30 min, and corneas were coated with 2% Methocel gel (OmniVision, Zurich, Switzerland) to prevent dryness. Fundus photography (FP) and fundus fluorescein angiography (FFA) were performed using a Micron III retinal imaging microscope (Phoenix Research Laboratories, San Ramon, CA, USA) with StreamPix software (version 6). FFA images were captured from the central retina centered on the optic disc with a field of view of 1.6 × 1.6 mm (36° × 36°) under standardized illumination and focus settings. Following FP, 10% sodium fluorescein was injected intraperitoneally, and FFA images were acquired using a 520 nm excitation filter.

### 2.7. Spectral-Domain Optical Coherence Tomography (OCT)

C57BL/6 mice were anesthetized, pupils dilated with 0.125% atropine (Sinphar, Taiwan), and 2% Methocel gel (OmniVision, Switzerland) applied to protect the cornea. Fundus and OCT imaging were performed using a Micron III retinal imaging microscope with an Image-Guided OCT System (Phoenix Research Laboratories, San Ramon, CA, USA). The foveal center was aligned with the center of the 1.6 × 1.6 mm field of view (36° × 36°), and white-light fundus images were acquired prior to OCT scans to ensure consistent positioning. For vascular imaging, 10% sodium fluorescein was injected intraperitoneally, and FFA images were obtained after complete vascular perfusion. Spectral-domain OCT images were collected in vertical or horizontal orientations using StreamPix software (version 6) and segmented with InSight XL. The retina was divided into three sublayers: inner (nerve fiber layer [NFL], ganglion cell layer [GCL], inner plexiform layer [IPL], inner nuclear layer [INL]); middle (outer plexiform layer [OPL], outer nuclear layer [ONL]); and outer (inner and outer segments of photoreceptors [IS/OS]). Thicknesses of the GCL, ONL, IS/OS, and retinal pigment epithelium (RPE) were measured for structural analysis. To evaluate longitudinal retinal changes, fundus photography (FP), FFA, and OCT images were obtained from the same animal at both baseline (Day 0) and post-intervention (Day 35). This within-subject design minimized inter-animal variability and enabled clearer visualization of treatment effects.

### 2.8. Hematoxylin and Eosin Stain (H&E Stain)

Eyes were fixed in 4% acidic formalin for 24 h, followed by 4% neutral-buffered formalin. To ensure anatomical consistency, the superior pole of each eyeball was marked immediately after enucleation, and all specimens were embedded in an identical orientation. Paraffin sections (3–5 µm) were cut along the superior–inferior axis through the optic nerve head to maintain comparable sampling across animals. Sections were stained with hematoxylin (1 min) and eosin (5 min), dehydrated through graded ethanol, cleared in xylene, and coverslipped. Retinal thickness was quantified 500 µm away from the optic nerve head on both the superior and inferior sides, which represent standardized and highly sensitive regions for detecting blue light–induced retinal degeneration. This approach provides reproducibility and minimizes inter-sample variation.

### 2.9. Antioxidant Enzyme Activity Levels Analysis

Blood samples were collected via cardiac puncture under anesthesia to minimize stress. Serum was isolated by centrifugation at 3000× *g* for 15 min at 4 °C and stored at –80 °C until analysis. The activities of antioxidant enzymes in serum were assessed using commercial enzyme activity assay kits for glutathione peroxidase (GPx) (Elabscience^®^, Houston, TX, USA) and catalase (CAT) (Biovision, Milpitas, CA, USA). Absorbance was measured using a microplate reader (BioTek PowerWave XS2, Winooski, VT, USA), and enzyme activities were calculated according to the manufacturers’ instructions.

### 2.10. Real-Time Quantitative Polymerase Chain Reaction (q-PCR)

Following animal sacrifice, total RNA was extracted from whole-eye and full-thickness colon tissues using TRIzol reagent (Thermo Fisher Scientific, Waltham, MA, USA) to obtain representative retinal and intestinal gene expression profiles. RNA was extracted according to the manufacturer’s protocol, quantified, and assessed for purity using a NanoDrop 1000 spectrophotometer (Thermo Scientific, Wilmington, DE, USA). Reverse transcription was performed with the iScript™ cDNA Synthesis Kit (Bio-Rad, Hercules, CA, USA). Quantitative real-time PCR (qPCR) was conducted using SYBR Green assays (Applied Biosystems, Foster City, CA, USA) on a LightCycler^®^ 96 System (Roche Diagnostics International AG, Rotkreuz, Switzerland). Target genes included inflammatory cytokines (*TNF-α*, *IL-1β*, *IL-6*), apoptosis-related markers (*Bax*, *Bcl-2*, *Caspase1*, *Caspase3*, *NLRP3*), and tight junction proteins (*Occludin*, *Claudin-1*, *ZO-1*), normalized to *GAPDH*. Relative expression was calculated using the 2^−ΔΔCt^ method. Primer sequences used in this study are provided in the Supplementary Materials (Table S1).

### 2.11. Gut Microbiota Analysis

Fresh fecal samples (~190 mg) were collected one day before sacrifice and stored at −80 °C. DNA was extracted using the QIAamp Fast DNA Stool Mini Kit (Qiagen, Germantown, MD, USA) and quantified with a NanoDrop 2000 spectrophotometer (Thermo Scientific, Wilmington, DE, USA), with purity confirmed by an OD_260/280 ratio of 1.8–2.0. The V3–V4 region of the bacterial 16S rRNA gene was amplified using primers 341F and 805R with Illumina adapter overhangs, indexed with the Nextera XT Index Kit (Illumina Inc., San Diego, CA, USA), and quality-checked with the Qsep100 Bio-Fragment Analyzer (BiOptic Inc., Taipei, Taiwan) [17]. Sequencing was performed on an Illumina MiSeq platform, and taxonomic classification was conducted using the SILVA v138 database [18].

Raw paired-end reads were quality-filtered, trimmed, and checked for chimeras before clustering into operational taxonomic units (OTUs) at ≥97% similarity. Alpha diversity was calculated using Observed and Chao1 indices; beta diversity was evaluated with unweighted and weighted principal coordinate analysis (PCoA). Differential taxa were identified using linear discriminant analysis effect size (LEfSe).

### 2.12. Statistical Analyses

Statistical analyses were performed using GraphPad Prism software (version 9.2.0). Data are presented as the mean ± standard error of the mean (SEM). One-way analysis of variance (ANOVA) followed by Tukey’s post hoc test was used for multiple group comparisons at a single time point. For parameters measured repeatedly in the same animals at different time points (Day 0 and Day 35), a mixed-effects model (restricted maximum likelihood method) was applied to account for within-subject variability and missing values. When sphericity was assumed, repeated-measures two-way ANOVA was performed as appropriate. A *p*-value of < 0.05 was considered statistically significant.

Correlation analyses of gut microbiota data were conducted using SPSS software (version 19.0) with Spearman’s rank correlation coefficient (ρ). Functional predictions of microbial metabolic pathways were performed using PICRUSt2, with statistical significance set at *p* < 0.05.

## 3. Results

### 3.1. Animal Experimental Design and Baseline Retinal Assessments

**Figure 2:**
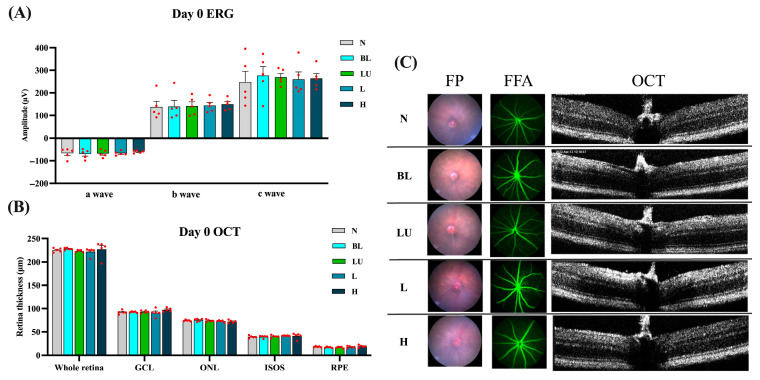
Baseline retinal structure and function before blue light exposure. (**A**) Quantification of Electroretinogram (ERG) results, including a-wave, b-wave, and c-wave amplitudes. (**B**,**C**) Retinal images from Day 0, including Fundus Photography (FP), Fundus Fluorescein Angiography (FFA), and Optical Coherence Tomography (OCT). (**B**) Quantification of all retinal layers from the OCT images, including the whole retina, Ganglion cell layer (GCL), outer nuclear layer (ONL), Inner segment/Outer segments (ISOS), retinal pigment epithelium (RPE). The red dots represent the distribution of the samples. (**C**) Retinal images were taken on Day 0. *n* = 5 per group. Data are presented as means ± SEM. Statistical analysis was performed using one-way ANOVA followed by Tukey’s post hoc test. Group labels: N (normal), BL (blue light), LU (lutein with blue light), L (*L. reuteri* Y7 10^8^ CFU/kg with blue light), H (*L. reuteri* Y7 10^9^ CFU/kg with blue light).

Baseline retinal structure and function were comprehensively assessed on Day 0, prior to blue light exposure, to ensure comparability among experimental groups. As shown in Figure 2, electroretinography (ERG) waveforms, including a-, b-, and c-waves, exhibited consistent amplitudes across the NC, BL, LU, L, and H groups, with no statistically significant differences (*p* > 0.05 for all comparisons), indicating equivalent baseline electrophysiological function. Quantitative spectral-domain optical coherence tomography (OCT) analysis revealed similar total retinal thickness and layer-specific measurements, including the ganglion cell layer (GCL), outer nuclear layer (ONL), inner segment–outer segment (IS/OS) junction, and retinal pigment epithelium (RPE), with no significant intergroup variation (*p* > 0.05). Representative multimodal imaging, comprising fundus photography (FP), fundus fluorescein angiography (FFA), and cross-sectional OCT, confirmed the absence of retinal pathology such as drusen-like deposits, atrophy, hemorrhage, or choroidal neovascularization (CNV). These findings collectively demonstrate that all animals began the experiment with structurally and functionally normal retinas, enabling valid post-intervention comparisons.

### 3.2. Retinal Function and Morphology After Intervention

**Figure 3:**
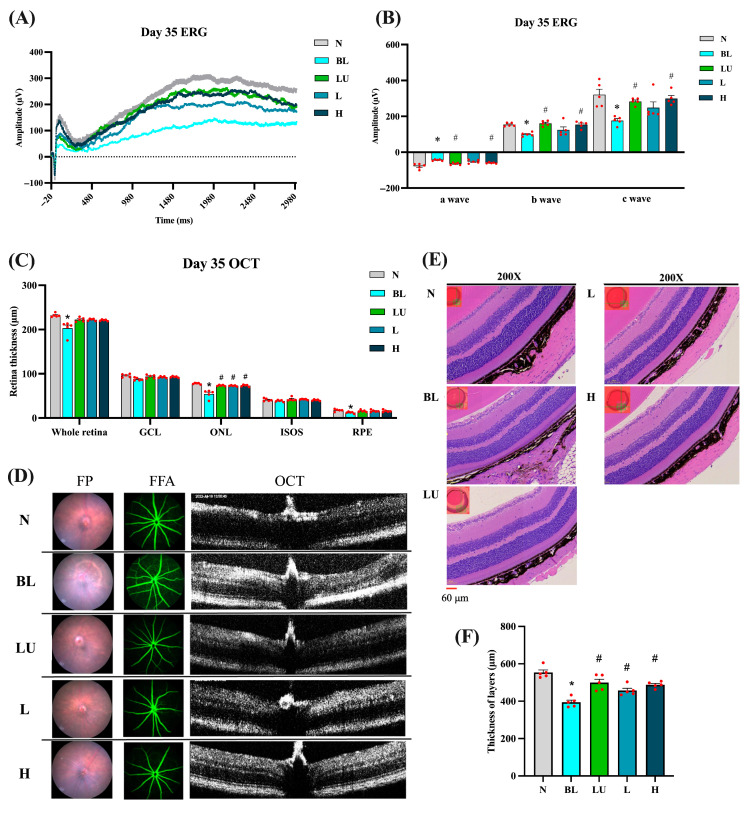
Retinal functional and morphological analysis after 35 days of intervention. (**A**) ERG showing representative a-wave, b-wave, and c-wave amplitudes. (**B**) Quantification of ERG results. (**C**) Quantification of retinal layers from OCT images. (**D**) Retinal images, including FP, FFA, and OCT. (**E**) Retinal images stained with H&E. (**F**) Quantification of retinal layers from H&E-stained sections. *n* = 5 per group. Data are presented as means ± SEM. * *p* < 0.05 for the BL group compared to the NC group; ^#^ *p* < 0.05 for LU, L, and H groups compared to the BL group. Statistical analysis was performed using one-way ANOVA followed by Tukey’s post hoc test. Group labels: N (normal), BL (blue light), LU (lutein with blue light), L (*L. reuteri* Y7 10^8^ CFU/kg with blue light), H (*L. reuteri* Y7 10^9^ CFU/kg with blue light). The red dots represent the distribution of the samples.

After 35 days of continuous blue light exposure, the blue light (BL) group exhibited pronounced retinal degeneration characteristic of an oxidative-stress–driven retinal-degeneration model with AMD-like features, whereas varying degrees of protection were observed in the lutein (LU) and low- (L) and high-dose (H) *Lactobacillus reuteri* Y7 groups (Figure 3). Electroretinography (ERG) traces (Figure 3A) showed marked reductions in a-, b-, and c-wave amplitudes in the BL group compared with the normal control (NC) group (*p* < 0.05 for all). The b-wave amplitudes were measured from the a-wave trough to the b-wave peak, in accordance with standard ERG procedures. The representative traces shown correspond to single animals with maximal responses, whereas the bar graphs depict group-averaged amplitudes, which explains the difference in absolute values between the two panels. Quantification (Figure 3B) confirmed significant decreases in all ERG components in the BL group, with partial preservation in the LU, L, and H groups (*p* < 0.05 vs. BL).

OCT analysis (Figure 3C) revealed a marked reduction in total retinal, outer nuclear layer (ONL), and retinal pigment epithelium (RPE) thickness in the BL group compared with the NC group (*p* < 0.05). Retinal thickness was assessed at standardized positions along the central retina, covering the superior and inferior regions adjacent to the optic nerve to ensure representative and reproducible measurements. All intervention groups (LU, L, and H) showed a significant recovery of ONL thickness relative to the BL group (*p* < 0.05), indicating strong structural protection. No significant differences were detected in the ganglion cell layer (GCL) or the inner segment–outer segment (IS/OS) junction.

Fundus photography and fluorescein angiography (Figure 3D) further visualized the retinal pathology: the BL group exhibited drusen-like deposits, mottled hyper/hypofluorescence, and focal atrophy, whereas these abnormalities were markedly reduced in the intervention groups, demonstrating consistent morphological protection.

Histological evaluation by hematoxylin and eosin (H&E) staining (Figure 3E) corroborated the OCT observations, revealing pronounced retinal thinning and disrupted laminar organization in the BL group, while the intervention groups retained well-defined retinal layering. Quantitative histomorphometric analysis (Figure 3F) confirmed a significant decrease in total retinal and ONL thickness in the BL group versus NC (*p* < 0.05), which was significantly reversed in all intervention groups (*p* < 0.05 vs. BL).

The absolute retinal thickness obtained from H&E was greater than that measured by SD-OCT. This apparent discrepancy arises from differences in anatomical reference boundaries, measurement orientation, and image-analysis criteria between the two techniques rather than from experimental error. Specifically, OCT-derived thickness represents the in vivo neurosensory retina (distance from the inner limiting membrane [ILM] to the outer border of the retinal pigment epithelium [RPE]), whereas histological measurements extend from the ILM to the outer border of the RPE–choroid complex, potentially including part of the underlying choroid. Such methodological differences, along with fixation-induced tissue swelling or oblique sectioning artifacts, can yield higher absolute values in H&E sections than in OCT measurements [19,20]. Importantly, despite the numerical differences, both approaches demonstrated the same directional trend (BL < LU/L/H ≈ NC), confirming the consistency and reliability of our structural assessments.

The absolute thickness measured by H&E was greater than OCT values, likely reflecting methodological differences between in vivo and ex vivo assessments.

Collectively, these imaging and histological findings demonstrate that both lutein and probiotic interventions provide substantial structural neuroprotection against blue light–induced retinal degeneration, primarily through preservation of ONL integrity.

### 3.3. Systemic Antioxidative Status After Intervention

As shown in Table 1, 35 days of continuous blue light exposure markedly reduced serum antioxidative enzyme activities in the blue light (BL) group compared with the normal control (NC) group. Catalase activity decreased from 18.48 ± 2.35 U/mL in NC to 2.28 ± 1.63 U/mL in BL, while glutathione peroxidase (GPx) activity declined from 1.49 ± 0.01 U/mL to 1.15 ± 0.07 U/mL (*p* < 0.05 for both), indicating a pronounced impairment of systemic antioxidative defense.

Supplementation with lutein (LU) or with *Lactobacillus reuteri* Y7 at either a low dose (L) or a high dose (H) significantly restored both catalase and GPx activities compared with BL (Tukey’s test, *p* < 0.0001 for all intervention groups vs. BL), with values approaching or exceeding those of the NC group. Although no significant differences were observed among the intervention groups, the LU group showed the numerically highest catalase activity, whereas the H group showed the numerically highest GPx activity, suggesting possible differential modulation of antioxidant pathways by lutein and probiotics. These findings indicate that lutein and probiotic supplementation effectively enhanced systemic antioxidative capacity and mitigated oxidative stress induced by chronic blue light exposure.

### 3.4. Inflammation-Related Gene Expression in the Eye

**Figure 4:**
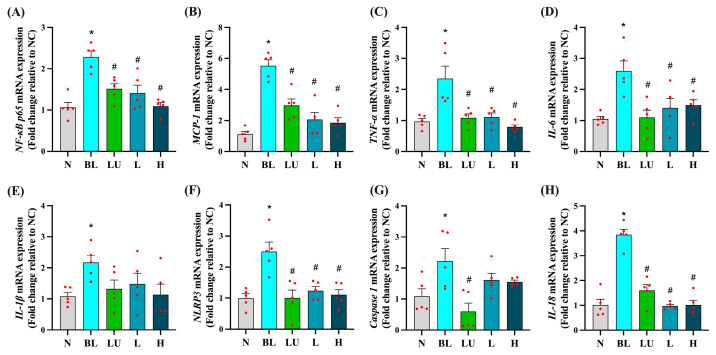
Ocular pro-inflammatory cytokine gene expression. (**A**) *NF-κB p65.* (**B**) *MCP-1*. (**C**) *TNF-α*. (**D**) *IL-6*. (**E**) *IL-1β*. (**F**) *NLRP3.* (**G**) *Caspase1*. (**H**) *IL18*. *n* = 5 per group. Data are presented as means ± SEM. * *p* < 0.05 for BL group compared to the NC group; ^#^ *p* < 0.05 for LU, L, and H groups compared to the BL group. Statistical analysis was performed using one-way ANOVA followed by Tukey’s post hoc test. Group labels: N (normal), BL (blue light), LU (lutein with blue light), L (*L. reuteri* Y7 10^8^ CFU/kg with blue light), H (*L. reuteri* Y7 10^9^ CFU/kg with blue light). The red dots represent the distribution of the samples.

Ocular mRNA expression profiles for inflammation-related genes are shown in Figure 4. In the BL group, *NF-κB p65*, *MCP-1*, *TNF-α*, *IL-6*, *IL-1β*, *NLRP3*, *Caspase-1*, and *IL-18* were all significantly upregulated compared with the NC group (*p* < 0.05 for each), indicating robust activation of pro-inflammatory pathways in the retina following blue light exposure. Supplementation with lutein (LU) or low (L) and high (H) doses of *Lactobacillus reuteri* Y7 significantly reduced *NF-κB p65*, *MCP-1*, *TNF-α*, *IL-6*, *NLRP3*, and *IL-18* expression compared with the BL group (*p* < 0.05). LU also significantly suppressed Caspase-1 expression, whereas the L and H groups showed a non-significant downward trend for this gene. For several pro-inflammatory markers, including *NF-κB p65*, *MCP-1*, *TNF-α*, and *IL-18*, probiotic intervention, particularly at the high dose, achieved reductions comparable to or slightly greater than those observed with lutein. These findings indicate that both lutein and probiotics can attenuate blue light-induced retinal inflammation, with probiotics demonstrating similar or superior effects for certain cytokines, while lutein exerts broader anti-inflammatory activity.

### 3.5. Apoptosis-Related Gene Expression in the Eye

**Figure 5:**
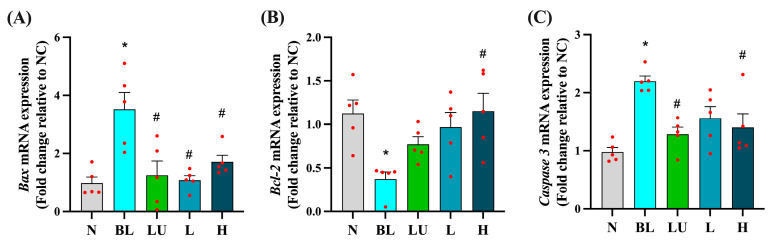
Ocular apoptosis-related gene expression. (**A**) *Bax.* (**B**) *Bcl2.* (**C**) *Caspase3*. *n* = 5 per group. Data are presented as means ± SEM. * *p* < 0.05 for the BL group compared to the NC group; ^#^ *p* < 0.05 for LU, L, and H groups compared to the BL group. Statistical analysis was performed using one-way ANOVA followed by Tukey’s post hoc test. Group labels: N (normal), BL (blue light), LU (lutein with blue light), L (*L. reuteri* Y7 10^8^ CFU/kg with blue light), H (*L. reuteri* Y7 10^9^ CFU/kg with blue light). The red dots represent the distribution of the samples.

As shown in Figure 5, chronic blue light exposure led to significant alterations in the expression of apoptosis-related genes in the eyes. The expression levels of the pro-apoptotic genes *Bax* and *Caspase-3* were significantly elevated in the blue light group (BL) compared to the normal control group (NC) (*p* < 0.05), indicating enhanced apoptotic signaling. Supplementation with lutein (LU) and high-dose probiotics (H) significantly reduced the expression of both *Bax* and *Caspase-3* compared to the BL group (*p* < 0.05), suggesting protective effects against retinal cell apoptosis. In contrast, the anti-apoptotic gene *Bcl-2* was significantly downregulated in the BL group (*p* < 0.05), reflecting impaired cell survival signaling. Among the intervention groups, only the high-dose probiotic group (H) showed a significant increase in *Bcl-2* expression relative to the BL group (*p* < 0.05), whereas the lutein and low-dose probiotic (L) groups did not exhibit significant changes in this marker.

These results suggest that high-dose probiotic supplementation offers stronger anti-apoptotic protection in the retina than either lutein or low-dose probiotics under conditions of blue light-induced retinal stress.

### 3.6. Tight Junction-Related Gene Expression in the Eye and Colon

**Figure 6:**
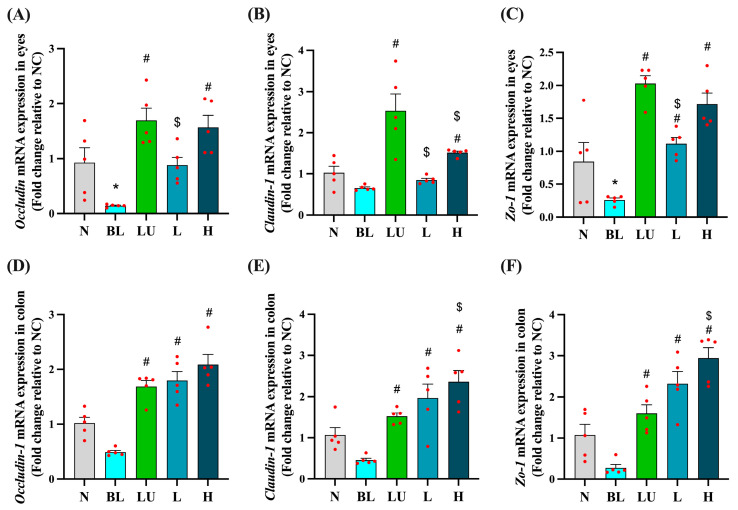
Ocular and colonic tight junction-related gene expression. (**A**–**C**) Eyes. (**D**–**F**) Colon. *n* = 5 per group. Data are presented as means ± SEM. * *p* < 0.05 for BL group compared to the NC group; ^#^ *p* < 0.05 for LU, L, and H groups compared to the BL group. $ *p* < 0.05 for L, and H groups compared to the LU group. Statistical analysis was performed using one-way ANOVA followed by Tukey’s post hoc test. Group labels: N (normal), BL (blue light), LU (lutein with blue light), L (*L. reuteri* Y7 10^8^ CFU/kg with blue light), H (*L. reuteri* Y7 10^9^ CFU/kg with blue light). The red dots represent the distribution of the samples.

As shown in Figure 6, tight junction-related gene expression was evaluated in both the eyes (Figure 6A–C) and the colon (Figure 6D–F) after different interventions. In the eye, the blue light exposure group (BL) showed significantly lower expression of *Occludin* and *ZO-1* compared to the normal control group (NC) (*p* < 0.05), while *Claudin-1* expression did not differ significantly between the BL and NC groups. However, supplementation with lutein (LU), low-dose probiotic (L), and high-dose probiotic (H) significantly increased the expression of all three genes compared to the BL group (*p* < 0.05). Additionally, the LU group showed significantly higher expression levels of *Occludin*, *ZO-1*, and *Claudin-1* than the L group (*p* < 0.05), and *Claudin-1* expression in the LU group was also significantly higher than in the H group (*p* < 0.05), indicating that lutein may provide stronger protection of retinal tight junctions than probiotics.

In the colon, although *Occludin*, *Claudin-1*, and *ZO-1* expression levels in the BL group were not significantly lower than in the NC group, all intervention groups showed significantly higher expression levels of these genes compared to the BL group (*p* < 0.05). Notably, the H group exhibited significantly higher expression of *Claudin-1* and *ZO-1* compared to the LU group (*p* < 0.05), suggesting that high-dose probiotic supplementation may offer greater enhancement of intestinal barrier integrity than lutein under blue light-induced systemic stress.

These results suggest that the interventions may support epithelial barrier integrity both locally in the retina and systemically in the gut, potentially implicating the gut–retina axis in blue light-induced pathology.

### 3.7. Gut Microbiota Composition and Diversity

**Figure 7:**
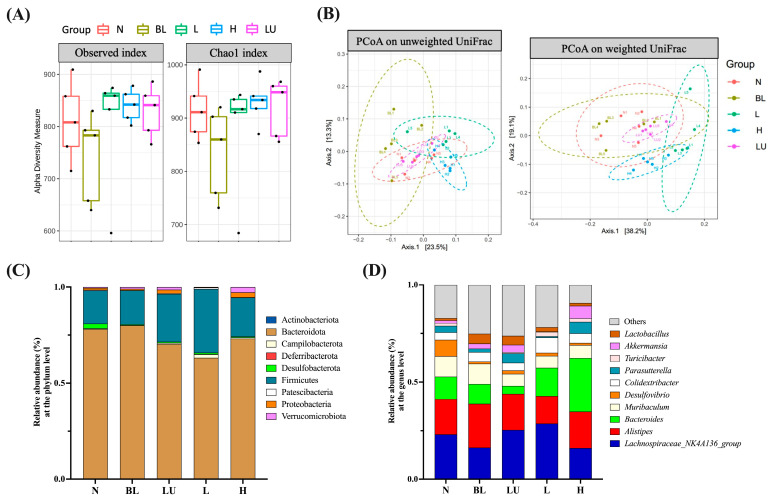
Gut microbiota composition and diversity across different groups. (**A**) Boxplots illustrating alpha diversity across different groups based on the Observed and Chao1 indices. The black dots within each bar represent the mean values, while the lines indicate the median values. (**B**) Principal Coordinate Analysis (PCoA) plots based on (**A**) unweighted UniFrac and (**B**) weighted UniFrac distance metrics for mouse fecal samples. Group clustering shows significant differences (Adonis, *p* < 0.05). Axes represent the percentage of data explained by each coordinate dimension. (**C**) Distribution of relative abundance of gut microbiota at the phylum level. (**D**) Distribution of relative abundance of gut microbiota at the genus level. *n* = 5 per group. Group labels: N (normal), BL (blue light), LU (lutein with blue light), L (*L. reuteri* Y7 10^8^ CFU/kg with blue light), H (*L. reuteri* Y7 10^9^ CFU/kg with blue light).

Preliminary analysis of gut microbiota was conducted through assessments of α diversity (enrichment), β diversity (composition), relative abundance, and Linear Discriminant Analysis Effect Size (LEfSe). As shown in Figure 7A, α diversity was evaluated using the Observed and Chao1 indices to estimate species richness within each group. Although numerical differences were noted among groups, no statistically significant differences were observed. β diversity, which reflects differences in microbial community composition among groups, was assessed using Principal Coordinate Analysis (PCoA) based on both unweighted and weighted UniFrac distances (Figure 7B). Clear group clustering patterns were observed in both analyses, with significant differences detected among groups (Adonis, *p* < 0.05), indicating that microbial community structures were markedly altered by different treatments.

In the relative abundance analysis, the top ten most abundant phyla (Figure 7C) were dominated by Bacteroidota and Firmicutes, followed by Actinobacteriota and Proteobacteria, which were the predominant phyla across all groups. At the genus level (Figure 7D), the ten most abundant genera included *Lachnospiraceae_NK4A136_group*, *Alistipes*, *Bacteroides*, *Muribaculum*, *Desulfovibrio*, *Colidextribacter*, *Parasutterella*, *Akkermansia*, *Turicibacter*, and *Lactobacillus*. These genera exhibited compositional changes across groups, suggesting potential modulations of gut microbiota by the respective interventions.

**Figure 8:**
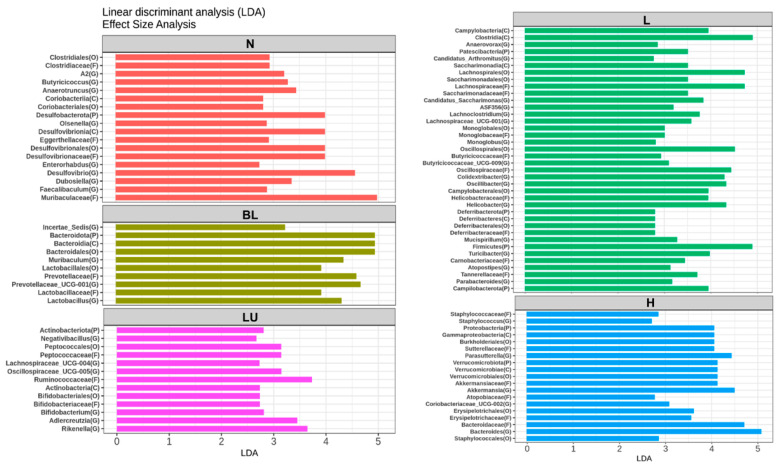
LEfSe analysis identifying gut bacterial markers that distinguish between groups. Only taxa with LDA scores >2.5 are shown on the right. *n* = 5 per group. Group labels: N (normal), BL (blue light), LU (lutein with blue light), L (*L. reuteri* Y7 10^8^ CFU/kg with blue light), H (*L. reuteri* Y7 10^9^ CFU/kg with blue light).

Figure 8 presents the results of the Linear Discriminant Analysis Effect Size (LEfSe), identifying gut microbial taxa that significantly differ between treatment groups. Only taxa with LDA scores greater than 2.5 are shown. In the normal (N) group, genera such as *Butyricicoccus*, *Anaerotruncus*, *Olsenella*, *Desulfovibrio*, *Enterorhabdus*, *Dubosiella*, and *Faecalibaculum* were significantly enriched, representing a baseline microbial profile typically associated with healthy physiological conditions. The blue light (BL) group showed significantly increased abundance of *Muribaculum*, *Lactobacillus*, and *Prevotellaceae_UCG-001*, suggesting that blue light exposure alone can induce shifts in gut microbial composition favoring these taxa. In the LU group (lutein combined with blue light), significant enrichment was observed in *Negativibacillus*, *Lachnospiraceae_UCG-004*, *Oscillospiraceae_UCG-005*, *Bifidobacterium*, *Adlercreutzia*, and *Rikenella*, suggesting a potential modulatory effect of lutein on the gut microbiota under oxidative stress conditions induced by blue light. The L group, which received low-dose probiotic supplementation (Y7 10^8^ CFU/kg), exhibited the most diverse and significantly altered genus-level composition. Enriched genera included *Anaerovorax*, *Candidatus_Arthromitus*, *Candidatus_Saccharimonas*, *ASF356*, *Lachnoclostridium*, *Lachnospiraceae_UCG-001*, *Monoglobus*, *Butyricicoccaceae_UCG-009*, *Colidextribacter*, *Oscillibacter*, *Helicobacter*, *Mucispirillum*, *Turicibacter*, *Atopostipes*, and *Parabacteroides*. These findings suggest that low-dose probiotic intervention may enhance microbial diversity and contribute to immune or metabolic modulation. In the H group, which received a high dose of probiotics (*L. reuteri* Y7 10^9^ CFU/kg), significantly enriched genera included *Staphylococcus*, *Parasutterella*, *Akkermansia*, *Coriobacteriaceae_UCG-002*, and *Bacteroides*. This microbial profile reflects a distinct gut microbiota restructuring potentially driven by higher probiotic intake, possibly linked to enhanced mucosal and metabolic functions.

Overall, LEfSe analysis reveals distinct microbial signatures across groups, demonstrating that both blue light exposure and interventions, particularly probiotics at varying doses, profoundly reshape the gut microbiota, which may in turn contribute to modulating systemic inflammation and retinal homeostasis under light-induced stress.

### 3.8. Correlation Between Gut Microbiota and Ocular Inflammation

**Figure 9:**
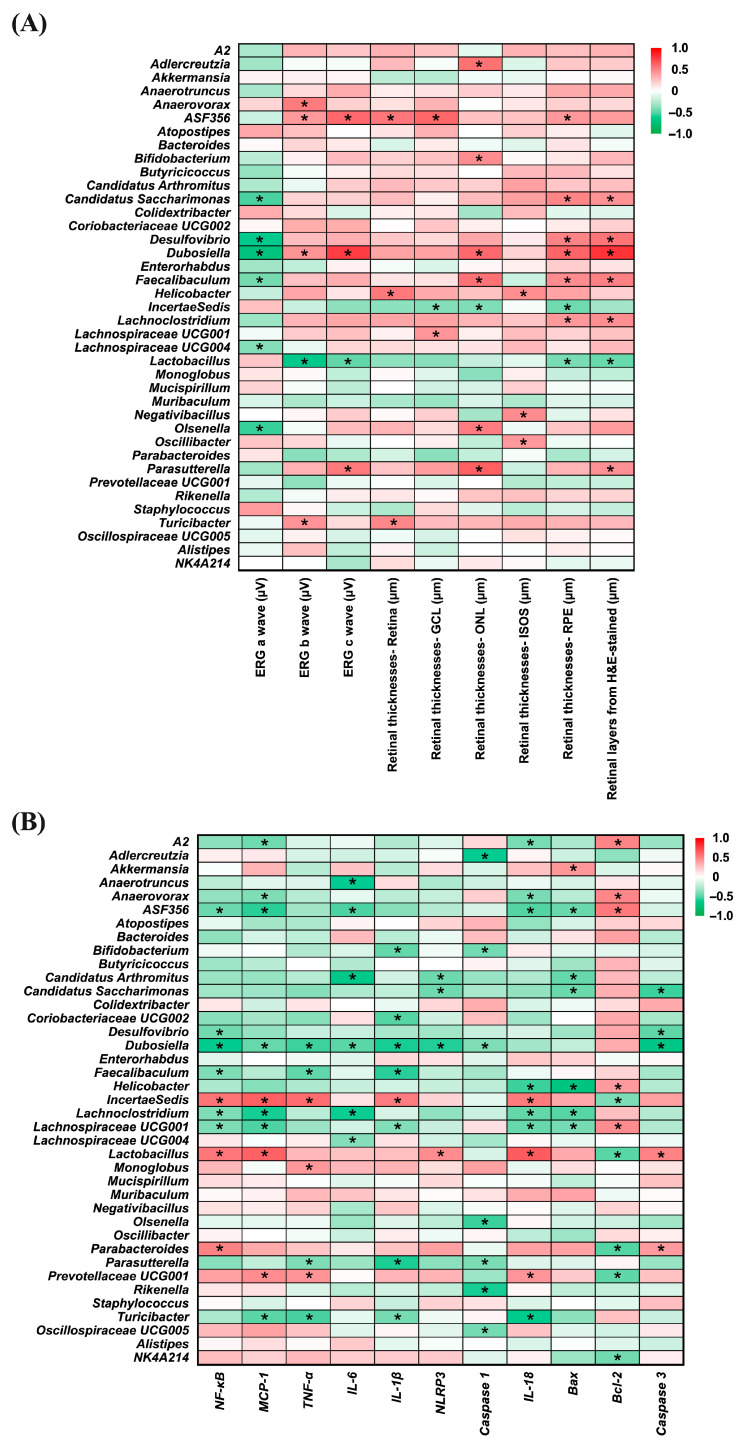
Correlation heatmaps of bacterial genera relative abundance. (**A**) Correlation with eye disease-related factors. (**B**) Correlation with inflammation-related factors. The color scale from green to red represents correlation coefficients ranging from −1 to 1. Statistically significant correlations are indicated by * *p* < 0.05. *n* = 5 per group. Statistical analysis was performed using correlation analysis. Group labels: N (normal), BL (blue light), LU (lutein with blue light), L (*L. reuteri* Y7 10^8^ CFU/kg with blue light), H (*L. reuteri* Y7 10^9^ CFU/kg with blue light).

Figure 9 presents heatmaps illustrating the correlations between gut bacterial genera and ocular disease-related parameters (Panel A), as well as inflammation- and apoptosis-related markers (Panel B). In Panel A, several genera, including *Candidatus_Saccharimonas *(*r* = −0.532; *p* = 0.006), *Desulfovibrio *(*r* = −0.628; *p* = 0.001), *Dubosiella *(*r* = −0.714; *p* < 0.001), *Faecalibaculum *(*r* = −0.439; *p* = 0.028), *Lachnospiraceae_UCG-004 *(*r* = −0.401; *p* = 0.047), and *Olsenella *(*r* = −0.562; *p* = 0.003) showed significant negative correlations with ERG a-wave amplitudes, suggesting an inverse association with photoreceptor activity. For the ERG b-wave, *Anaerovorax*, *ASF356 *(*r* = 0.524; *p* = 0.007), *Dubosiella *(*r* = 0.426; *p* = 0.034), and *Turicibacter *(*r* = 0.430; *p* = 0.032) were positively correlated, whereas *Lactobacillus *(*r* = −0.652; *p* < 0.001), exhibited a significant negative correlation. A similar pattern was observed for the ERG c-wave, where *ASF356 *(*r* = 0.589; *p* = 0.002), *Dubosiella *(*r* = 0.753; *p* < 0.001), and *Parasutterella *(*r* = 0.525; *p* = 0.007) were positively correlated, while *Lactobacillus *(*r* = −0.483; *p* = 0.015) again showed a significant negative correlation. Positive correlations with whole retinal thickness were found for *ASF356 *(*r* = 0.535; *p* = 0.006), *Helicobacter *(*r* = 0.532; *p* = 0.006), and *Turicibacter *(*r* = 0.479; *p* = 0.015). In the ganglion cell layer (GCL), *ASF356 *(*r* = 0.598; *p* = 0.002), and *Lachnospiraceae_UCG-001 *(*r* = 0.420; *p* = 0.037) showed positive associations, whereas *Incertae Sedis *(*r* = −0.401; *p* = 0.047), was negatively correlated. For the outer nuclear layer (ONL), genera such as *Adlercreutzia *(*r* = 0.561; *p* = 0.004), *Bifidobacterium *(*r* = 0.451; *p* = 0.024), *Dubosiella *(*r* = 0.589; *p* = 0.002), *Faecalibaculum *(*r* = 0.553; *p* = 0.004), *Olsenella *(*r* = 0.514; *p* = 0.009), and *Parasutterella *(*r* = 0.622; *p* = 0.001) were positively correlated, in contrast to *Incertae Sedis *(*r* = −0.409; *p* = 0.042), which exhibited a significant negative correlation. Increased thickness of the ISOS layer was positively associated with *Helicobacter *(*r* = 0.447; *p* = 0.025), *Negativibacillus *(*r* = 0.453; *p* = 0.023), and *Oscillibacter *(*r* = 0.405; *p* = 0.045), while in the photoreceptor layer (PRE), positive correlations were observed with *ASF356 *(*r* = 0.411; *p* = 0.041), *Candidatus_Saccharimonas *(*r* = 0.484; *p* = 0.014), *Desulfovibrio *(*r* = 0.488; *p* = 0.013), *Dubosiella *(*r* = 0.608; *p* = 0.001), *Faecalibaculum *(*r* = 0.453; *p* = 0.023), and *Lachnoclostridium *(*r* = 0.408; *p* = 0.043), and negative correlations with *Incertae Sedis *(*r* = −0.452; *p* = 0.023) and *Lactobacillus *(*r* = −0.426; *p* = 0.034). Moreover, H&E-stained retinal sections revealed that *Candidatus_Saccharimonas *(*r* = 0.431; *p* = 0.032), *Desulfovibrio *(*r* = 0.551; *p* = 0.004), *Dubosiella *(*r* = 0.785; *p* < 0.001), *Faecalibaculum *(*r* = 0.500; *p* = 0.011), *Lachnoclostridium *(*r* = 0.445; *p* = 0.026), and *Parasutterella *(*r* = 0.439; *p* = 0.028) were positively associated with retinal thickness, whereas *Lactobacillus *(*r* = −0.462; *p* = 0.020) showed a consistent negative correlation.

In Panel B, several bacterial genera displayed notable correlations with inflammation- and apoptosis-related markers. *ASF356 *(*r* = −0.460; *p* = 0.021), *Desulfovibrio *(*r* = −0.445; *p* = 0.026), *Dubosiella *(*r* = −0.620; *p* = 0.001), *Faecalibaculum *(*r* = −0.412; *p* = 0.041), *Lachnoclostridium *(*r* = −0.415; *p* = 0.039), and *Lachnospiraceae_UCG-001 *(*r* = −0.412; *p* = 0.041) showed significant negative correlations with NF-κB, whereas *Incertae Sedis *(*r* = 0.549; *p* = 0.004), *Lactobacillus *(*r* = 0.539; *p* = 0.005), and *Parabacteroides *(*r* = 0.495; *p* = 0.012) were positively correlated. For MCP-1, genera including *A2 *(*r* = −0.449; *p* = 0.024), *Anaerovorax *(*r* = −0.412; *p* = 0.041), *ASF356 *(*r* = −0.579; *p* = 0.002), *Dubosiella *(*r* = −0.468; *p* = 0.018), *Lachnoclostridium *(*r* = −0.595; *p* = 0.002), *Lachnospiraceae_UCG-001 *(*r* = −0.521; *p* = 0.008), and *Turicibacter *(*r* = −0.500; *p* = 0.011) were negatively correlated, while *Incertae Sedis *(*r* = 0.625; *p* = 0.001), *Lactobacillus *(*r* = 0.618; *p* = 0.001) and *Prevotellaceae_UCG001 *(*r* = 0.456; *p* = 0.022) were positively associated. In terms of TNF-α, negative correlations were observed with *Dubosiella *(*r* = −0.552; *p* = 0.004), *Faecalibaculum *(*r* = −0.481; *p* = 0.015), *Parasutterella *(*r* = −0.414; *p* = 0.040), and *Turicibacter *(*r* = −0.536; *p* = 0.006), while *Incertae Sedis *(*r* = 0.575; *p* = 0.003), *Monoglobus *(*r* = 0.410; *p* = 0.042), and *Prevotellaceae_UCG001 *(*r* = 0.425; *p* = 0.034) showed positive associations.

Regarding IL-6, *Anaerotruncus *(*r* = −0.617; *p* = 0.001), *ASF356 *(*r* = −0.527; *p* = 0.007), *Candidatus Arthromitus *(*r* = −0.662; *p* < 0.001), *Dubosiella *(*r* = −0.497; *p* = 0.012), *Lachnoclostridium *(*r* = −0.598; *p* = 0.002), and *Lachnospiraceae_UCG-004 *(*r* = −0.396; *p* = 0.050) exhibited negative correlations. Negative associations with IL-1β were found for *Bifidobacterium *(*r* = −0.474; *p* = 0.017), *Coriobacteriaceae_UCG002 *(*r* = −0.508; *p* = 0.010), *Dubosiella *(*r* = −0.595; *p* = 0.002), *Faecalibaculum *(*r* = −0.578; *p* = 0.002), *Lachnospiraceae_UCG-001 *(*r* = −0.405; *p* = 0.045), *Parasutterella *(*r* = −0.592; *p* = 0.002), and *Turicibacter *(*r* = −0.428; *p* = 0.033), whereas *Incertae Sedis *(*r* = 0.515; *p* = 0.008) was positively correlated. For NLRP3, *Candidatus Arthromitus *(*r* = −0.457; *p* = 0.022), *Candidatus_Saccharimonas *(*r* = −0.450; *p* = 0.024), and *Dubosiella *(*r* = −0.584; *p* = 0.002) were negatively correlated, in contrast to *Lactobacillus *(*r* = 0.457; *p* = 0.022), which showed a significant positive correlation.

Negative correlations with Caspase-1 were identified for *Adlercreutzia *(*r* = −0.611; *p* = 0.001), *Bifidobacterium *(*r* = −0.438; *p* = 0.028), *Dubosiella *(*r* = −0.412; *p* = 0.041), *Olsenella *(*r* = −0.554; *p* = 0.004), *Parasutterella *(*r* = −0.418; *p* = 0.038), *Rikenella *(*r* = −0.596; *p* = 0.002), and *Oscillospiraceae_UCG005 *(*r* = −0.434; *p* = 0.030). Several genera—including *A2 *(*r* = −0.430; *p* = 0.032), *Anaerovorax *(*r* = −0.422; *p* = 0.035), *ASF356 *(*r* = −0.514; *p* = 0.009), *Helicobacter *(*r* = −0.557; *p* = 0.004), *Lachnoclostridium *(*r* = −0.480; *p* = 0.015), *Lachnospiraceae_UCG-001 *(*r* = −0.471; *p* = 0.018), and *Turicibacter *(*r* = −0.624; *p* = 0.001)—were negatively associated with IL-18, whereas *Incertae Sedis *(*r* = 0.525; *p* = 0.007), *Lactobacillus *(*r* = 0.642; *p* = 0.001), and *Prevotellaceae_UCG001 *(*r* = 0.419; *p* = 0.037) showed positive correlations. Additionally, *Akkermansia *(*r* = 0.413; *p* = 0.040) was positively correlated with Bax, while *ASF356 *(*r* = −0.466; *p* = 0.019), *Candidatus Arthromitus *(*r* = −0.470; *p* = 0.018), *Candidatus_Saccharimonas *(*r* = −0.462; *p* = 0.020), *Helicobacter *(*r* = −0.696; *p* < 0.001), *Lachnoclostridium *(*r* = −0.503; *p* = 0.010), and *Lachnospiraceae_UCG-001 *(*r* = −0.432; *p* = 0.031) were negatively correlated.

Positive correlations with the anti-apoptotic marker Bcl-2 were found in *A2 *(*r* = 0.489; *p* = 0.013), *Anaerovorax *(*r* = 0.477; *p* = 0.016), *ASF356 *(*r* = 0.531; *p* = 0.006), *Helicobacter *(*r* = 0.397; *p* = 0.049), and *Lachnospiraceae_UCG-001 *(*r* = −0.456; *p* = 0.022) whereas *Incertae Sedis *(*r* = −0.419; *p* = 0.037), *Lactobacillus *(*r* = −0.499; *p* = 0.011), *Parabacteroides *(*r* = −0.515; *p* = 0.008), *Prevotellaceae_UCG001 *(*r* = −0.488; *p* = 0.013), and *NK4A214 *(*r* = −0.444; *p* = 0.026) showed negative associations. Finally, *Candidatus_Saccharimonas *(*r* = −0.555; *p* = 0.004), *Desulfovibrio *(*r* = −0.492; *p* = 0.012), and *Dubosiella *(*r* = −0.674; *p* < 0.001) exhibited negative correlations with Caspase-3, while *Lactobacillus *(*r* = 0.495; *p* = 0.012) and *Parabacteroides *(*r* = 0.397; *p* = 0.050) were positively correlated.

These correlation patterns support the involvement of specific gut bacteria in modulating retinal health through regulation of inflammation and apoptosis, highlighting the relevance of the gut–retina axis in light-induced retinal injury.

## 4. Discussion

In this study, we demonstrated that oral administration of *Lactobacillus reuteri* Y7 effectively attenuates blue light (BL) induced retinal degeneration in a murine model. The protective effects of Y7 were evidenced by preservation of retinal morphology and electrophysiological function, as assessed by OCT imaging and ERG analysis. Mechanistically, Y7 supplementation significantly enhanced systemic antioxidant enzyme activities, including catalase and glutathione peroxidase (GPx), which are critical in mitigating reactive oxygen species (ROS) accumulation. In addition to its antioxidant role, Y7 downregulated inflammatory and pro-apoptotic genes in retinal tissues, while concurrently upregulating tight junction protein expression in both the eye and colon. Furthermore, Y7 administration induced dose-dependent shifts in gut microbiota composition, and correlation analyses revealed significant associations between specific bacterial taxa and retinal or inflammatory markers. Together, these findings suggest that *L. reuteri* Y7 confers retinal protection via a multi-faceted mechanism, involving antioxidant defense, immune modulation, epithelial barrier preservation, and gut–retina axis regulation.

Age-related macular degeneration (AMD) is currently recognized as the third leading cause of blindness worldwide [21]. Although pharmacological treatments are available for advanced wet AMD, effective therapeutic strategies for the more prevalent dry AMD remain limited, resulting in a substantial gap in the management of early-stage disease. Among environmental risk factors, chronic exposure to short-wavelength blue light has been shown to induce oxidative stress, inflammation, immune dysregulation, and apoptosis in ocular tissues [22], all of which contribute to the progression of retinal degeneration. Chronic blue light exposure is a well-established inducer of retinal phototoxicity, primarily through the generation of reactive oxygen species (ROS) and subsequent oxidative damage to photoreceptors and the retinal pigment epithelium (RPE) [4,23]. In our model, 35 days of continuous blue light exposure resulted in severe functional and structural degeneration with dry AMD-like features [4,23,24,25,26,27], consistent with previous reports in murine blue light injury models [28,29].

Although phototoxicity-based models do not fully replicate the complex and multifactorial etiology of AMD, they reproduce several hallmark pathological features, including outer nuclear layer (ONL) thinning, RPE damage, drusen-like deposits, and functional decline. These changes involve oxidative and inflammatory pathways that are also implicated in AMD pathogenesis. In particular, recruitment of macrophages and secretion of inflammatory cytokines in response to local oxidative stress in the macular area is a well-recognized pathogenetic mechanism for AMD development [30,31], indicating that chronic blue light–induced oxidative injury may activate downstream inflammatory and neurodegenerative cascades similar to those observed in AMD.

Dietary supplementation with antioxidant-rich nutraceuticals has been extensively studied for retinal protection, particularly in the context of AMD. Lutein and zeaxanthin, which are macular carotenoids that accumulate in the retina, help filter blue light, quench singlet oxygen, and neutralize reactive oxygen species [32,33]. The AREDS2 trial evaluated supplementation with lutein (10 mg) and zeaxanthin (2 mg) and found modest benefits in slowing progression to late AMD, particularly among individuals with low baseline intake or those not receiving beta-carotene [34,35]. However, responses to supplementation vary depending on nutritional status, diet, and genetic background, indicating the need for complementary or alternative approaches.

In response to these limitations, emerging evidence has identified probiotics as promising non-invasive interventions for retinal protection under oxidative stress conditions. For instance, *Lactobacillus paracasei* KW3110 has been reported to attenuate photoreceptor apoptosis and suppress retinal inflammation in light-induced injury models [7]. Similarly, *Lactobacillus fermentum* NS9, particularly when combined with anthocyanin-rich extracts, has been shown to preserve retinal structure and improve oxidative stress markers in sodium iodate-induced degeneration models [10]. These findings suggest that probiotics may confer retinal resilience through mechanisms distinct from, yet potentially complementary to, those of traditional antioxidant nutraceuticals.

Moreover, a recent meta-analysis demonstrated that probiotic supplementation significantly reduces oxidative stress biomarkers, including malondialdehyde (MDA), while enhancing total antioxidant capacity (TAC) and glutathione levels across both clinical and preclinical settings [36]. These findings support the systemic antioxidant potential of probiotics beyond the gastrointestinal tract. Consistent with these findings, our study revealed that oral administration of *Lactobacillus reuteri* Y7 significantly increased the activities of systemic antioxidant enzymes, notably catalase and glutathione peroxidase (GPx), in mice exposed to blue light (Table 1). These enzymes play a crucial role in neutralizing reactive oxygen species (ROS), the accumulation of which is a central contributor to retinal oxidative damage. The enhanced antioxidant capacity observed in Y7-treated mice was accompanied by preserved retinal electrophysiological responses (ERG, Figure 3A,B) and maintenance of retinal structural integrity (OCT and H&E staining, Figure 3C–F), underscoring the critical role of oxidative stress mitigation in sustaining visual function under phototoxic conditions.

In addition to its pro-oxidative effects, blue light exposure activates a cascade of inflammatory mediators that exacerbate retinal injury. In our study, blue light exposure significantly upregulated the expression of *NF-κB p65*, *TNF-α*, *IL-6*, *IL-1β*, *NLRP3*, and *IL-18* in retinal tissue (Figure 4), reflecting a chronic inflammatory state associated with phototoxic stress. Notably, Y7 supplementation, particularly at high doses, significantly suppressed these mediators, suggesting an attenuation of retinal innate immune activation. This observation aligns with earlier reports showing that *Lactiplantibacillus plantarum* EP21 and *Lacticaseibacillus rhamnosus* downregulate *NF-κB* signaling and pro-inflammatory cytokines in ocular models [37]. Concurrently, we observed that Y7 also influenced apoptosis-related gene expression, with downregulation of *Bax* and *Caspase-3* and upregulation of *Bcl-2* in the high-dose group (Figure 5). These changes indicate improved cell survival signaling and reduced photoreceptor vulnerability. Similar anti-apoptotic effects have been reported for *Limosilactobacillus fermentum* HY7302, which mitigated ROS-induced cell death by modulating the Bax/Bcl-2 and caspase pathways in conjunctival epithelial cells [38]. Collectively, these results suggest that Y7 confers dual protection by suppressing inflammatory responses and preserving retinal cell viability.

Disruption of epithelial barriers has been increasingly recognized as a contributing factor in the pathogenesis of both ocular and systemic inflammatory diseases. In our study, blue light exposure significantly downregulated the expression of the tight junction–associated genes *Occludin*, *ZO-1*, and *Claudin-1* in both retinal and colonic tissues, indicating a loss of epithelial barrier integrity (Figure 6). This is consistent with prior in vitro studies demonstrating that blue LED exposure leads to dose-dependent TJ dysfunction in ARPE-19 cells, even before overt cell death occurs [6]. Additionally, A2E-loaded RPE models exhibited discontinuous ZO-1 localization and reduced transepithelial electrical resistance (TEER) upon blue light exposure, further confirming barrier breakdown [39]. Moreover, Claudin-5, a key component of the inner blood–retinal barrier, was shown to be rapidly downregulated in retinal endothelial cells following blue light exposure, resulting in increased paracellular permeability [36].

Importantly, Y7 supplementation effectively restored TJ gene expression in both the eye and colon. Notably, the high-dose Y7 group exhibited even higher expression levels of *Claudin-1* and *ZO-1* in the colon compared to the lutein-treated group (Figure 6F), suggesting systemic barrier-supportive effects. These results are in line with previous evidence that probiotics such as *Lactobacillus plantarum* MB452 and multi-strain formulations can enhance TEER and upregulate TJ proteins in intestinal epithelial models, while preventing inflammation-induced barrier dysfunction [40,41,42]. Reviews have further confirmed the broad capacity of probiotics to promote TJ reassembly, mucosal regeneration, and epithelial resilience under stress conditions [43,44].

The gut–retina axis has emerged as a key framework linking intestinal microbial composition to ocular health, particularly through modulation of systemic immune responses, oxidative stress, and metabolic signaling [45,46]. Disruption of gut homeostasis can promote systemic inflammation, alter circulating metabolites, and compromise the blood–retina barrier, thereby facilitating retinal degeneration [47,48].

Our LEfSe analysis revealed distinct gut microbial signatures across experimental groups, suggesting that both blue light exposure and subsequent interventions significantly modulate gut microbial composition, potentially influencing systemic inflammation and retinal homeostasis (Figure 8 and Figure 9). In the normal control group, enrichment of genera such as *Butyricicoccus*, *Anaerotruncus*, *Olsenella*, and *Faecalibaculum* is consistent with a healthy microbial profile enriched in butyrate-producing bacteria, which are known to support intestinal barrier integrity and exert anti-inflammatory effects through short-chain fatty acid (SCFA) signaling [47,49]. Notably, *Butyricicoccus* has been reported as reduced in diabetic retinopathy patients, and SCFA-mediated mechanisms, such as protection of the blood–retina barrier and suppression of inflammatory cascades, have been implicated in ameliorating early neurovascular unit damage in the diabetic retina [12,50,51,52,53]. *Anaerotruncus* is enriched in neovascular age-related macular degeneration and may be linked to pro-inflammatory signaling [51,54,55]. While *Olsenella* has limited direct evidence linking it to retinal health, it forms part of the commensal gut microbiota and may contribute to systemic immune regulation [56]. *Faecalibaculum*, though less studied in ocular disease, belongs to SCFA-producing taxa that potentially support gut and retinal homeostasis [55,57]. Collectively, these taxa may represent protective microbial signatures that help counteract inflammation and preserve retinal function.

In contrast, blue light exposure alone (BL group) was associated with increased abundance of *Muribaculum*, *Lactobacillus*, and *Prevotellaceae_UCG-001*. Although certain *Lactobacillus* species are generally considered beneficial, their overrepresentation under stress conditions has been linked to gut dysbiosis and altered mucosal immunity [58]. Similarly, *Prevotellaceae* enrichment has been associated with pro-inflammatory mucosal responses and metabolic disturbances [59,60], suggesting that chronic light-induced stress may favor microbial communities with inflammatory potential.

Lutein supplementation under blue light exposure (LU group) selectively enriched *Bifidobacterium*, *Adlercreutzia*, and *Rikenella*, alongside members of the Lachnospiraceae and Oscillospiraceae families. *Bifidobacterium* is well-documented for enhancing gut barrier integrity, producing acetate, and modulating immune tolerance [61]. This suggests that lutein’s protective role may extend beyond its direct antioxidant effects to include microbiota-mediated modulation of host immunity.

Low-dose *Lactobacillus reuteri* Y7 (L group) induced the most extensive genus-level shifts, characterized by the enrichment of *Parabacteroides*, *Turicibacter*, and *Monoglobus. Parabacteroides* has been shown to exert anti-inflammatory effects and facilitate bile acid metabolism, thereby indirectly modulating oxidative stress and metabolic signaling through activation of FXR/TGR5 and downstream Nrf2 antioxidant pathways, as well as via SCFA-mediated suppression of NF-κB activation [62,63,64,65]. In addition, outer membrane vesicles derived from *P. distasonis* have been reported to enhance IL-10 production and reduce pro-inflammatory cytokine release [66]. *Turicibacter* participates in tryptophan metabolism, generating indole derivatives such as indolepropionic acid, which acts as an antioxidant and modulates immune responses through AhR signaling [67,68], and it also influences gut-derived serotonin production, potentially affecting both gut–brain and gut–retina communication [69]. *Monoglobus* is a recently described genus specialized in the degradation of dietary pectin and other complex polysaccharides, producing SCFAs such as acetate and propionate [70,71]. These metabolites can serve as substrates for cross-feeding butyrate-producing bacteria, thereby indirectly enhancing intestinal barrier integrity and reducing systemic inflammation [72,73,74], which may in turn support retinal homeostasis [75,76]. The concurrent enrichment of multiple *Lachnospiraceae* members and other SCFA producers (e.g., *Butyricicoccaceae_UCG-009* and *Oscillibacter*) further supports the role of low-dose probiotic intervention in restoring metabolic and immune homeostasis [77,78]. Collectively, these metabolic features suggest that the enriched taxa may contribute to retinal homeostasis by attenuating inflammation, oxidative stress, and vascular dysregulation [75,78].

High-dose Y7 (H group) was associated with the enrichment of *Akkermansia*, *Parasutterella*, and *Bacteroides*. *Akkermansia muciniphila* is a well-known mucin-degrading bacterium that resides in the mucus layer and plays a pivotal role in maintaining epithelial barrier integrity [79,80,81]. By utilizing mucin as a carbon and nitrogen source, *A. muciniphila* promotes mucus turnover, upregulates the expression of tight junction proteins (e.g., occludin, claudins, and ZO-1), and reduces gut permeability [79,81]. It also produces short-chain fatty acids (SCFAs), particularly acetate and propionate, which activate G-protein–coupled receptors (GPR41/43) and suppress NF-κB–mediated inflammatory pathways [81]. In addition, A. muciniphila releases outer membrane proteins (e.g., Amuc_1100) that interact with Toll-like receptor 2 (TLR2), thereby enhancing anti-inflammatory cytokine production and improving host metabolic parameters, including glucose tolerance and lipid metabolism [82]. *Parasutterella*, a member of the *Betaproteobacteria*, is involved in bile acid and cholesterol metabolism [83]. It contributes to primary bile acid deconjugation and modulates the composition of the bile acid pool, thereby influencing farnesoid X receptor (FXR) and Takeda G-protein–coupled receptor 5 (TGR5) signaling [84]. Activation of these bile acid receptors is associated with improved lipid homeostasis, attenuation of hepatic steatosis, and systemic anti-inflammatory effects [84]. Moreover, *Parasutterella* produces succinate, a key intermediate of the tricarboxylic acid (TCA) cycle, which functions as a signaling molecule regulating intestinal immune responses and epithelial oxygen consumption [85]. *Bacteroides* is one of the most dominant genera in the human gut microbiota and is specialized in degrading complex dietary polysaccharides, such as resistant starch, pectin, and hemicellulose, through a diverse repertoire of carbohydrate-active enzymes (CAZymes) [86]. This fermentation process yields SCFAs, primarily acetate and propionate, which support energy harvest, reinforce intestinal barrier function, and modulate immune responses [87]. In addition, *Bacteroides* species can alter bile acids via bile salt hydrolase activity, potentially affecting FXR/TGR5 signaling and downstream anti-inflammatory and metabolic pathways [88]. Certain Bacteroides-derived capsular polysaccharides, such as polysaccharide A (PSA), have also been shown to promote regulatory T cell (Treg) expansion and IL-10 production, thereby mitigating intestinal and systemic inflammation [89]. Collectively, the enrichment of *Akkermansia*, *Parasutterella*, and *Bacteroides* under high-dose probiotic administration may synergistically enhance mucosal resilience, fortify intestinal barrier function, and optimize metabolic regulation through complementary effects on mucus layer maintenance, SCFA production, bile acid signaling, and immune modulation.

Taken together, these findings indicate a dose-dependent modulation of the gut microbiota by *L. reuteri* Y7. Low-dose intervention appears to favor microbial diversity and the enrichment of SCFA-producing commensals, which may confer anti-inflammatory and metabolic benefits. In contrast, high-dose intervention promotes taxa involved in mucosal barrier reinforcement and metabolic regulation. Considering the emerging evidence supporting the existence of a gut–retina axis [11], such microbial shifts may play a crucial role in mitigating oxidative stress and inflammatory retinal damage during chronic blue light exposure.

Lutein, a well-known dietary antioxidant, provided substantial retinal protection in this model. Notably, high-dose administration of *L. reuteri* Y7 matched or even outperformed lutein in several key parameters, including retinal layer preservation (Figure 3C,F), suppression of inflammation (Figure 4), and maintenance of gut barrier integrity (Figure 6). These findings position probiotics as viable alternatives or complementary interventions to existing antioxidant therapies. While previous studies have demonstrated systemic antioxidant effects of probiotics, few have directly examined their impact on ocular tissues under phototoxic stress. The present study addresses this gap, demonstrating that retinal protection can be achieved through systemic microbial modulation.

Both lutein and probiotic interventions may act through complementary antioxidant and anti-inflammatory mechanisms. Lutein primarily functions as a direct free-radical scavenger within ocular tissues, whereas *L. reuteri* Y7 enhances systemic antioxidant defenses and modulates inflammatory signaling through gut-mediated pathways. These parallel actions suggest that a combined lutein and Y7 regimen could yield additive or even synergistic protection against blue-light–induced retinal degeneration by jointly targeting local oxidative stress and systemic metabolic regulation. Future investigations should evaluate such combined interventions to clarify whether their effects are synergistic or additive and to guide the development of multi-target strategies integrating ocular and systemic protection.

This study was limited by a small sample size (*n* = 5 per group), although the observed differences were statistically significant. Future studies should therefore assess long-term efficacy and safety in larger cohorts. In addition, the use of a murine model poses inherent limitations, as mice possess nocturnal vision and lower natural protection against blue light than humans. These physiological differences may influence the magnitude and kinetics of retinal injury; hence, the results should be interpreted with caution when extending them to human conditions.

The mechanistic link between microbial metabolites and ocular outcomes also warrants further exploration, potentially through metabolomics and fecal microbiota transplantation. Additional studies should determine whether live probiotic strains or postbiotics are more effective for clinical application. Moreover, future research should benchmark Y7 against other antioxidant comparators, particularly zeaxanthin and anthocyanins, using standardized dosing paradigms to facilitate cross-study synthesis.

In conclusion, *Lactobacillus reuteri* Y7 protects against blue light–induced retinal damage through mechanisms involving systemic antioxidant defense, retinal anti-inflammatory activity, regulation of apoptosis, preservation of tight junction integrity, and modulation of the gut microbiota. This integrated protective effect underscores the potential of microbiota-targeted strategies as non-invasive interventions for retinal degenerative diseases, particularly in the context of widespread digital screen exposure. Overall, these findings align with an antioxidant-centered therapeutic framework that is highly relevant to the scope of Antioxidants.

## 5. Conclusions

Oral administration of *Lactobacillus reuteri* Y7 mitigated blue light–induced retinal degeneration in mice by preserving retinal function and morphology while enhancing systemic antioxidant capacity and dampening inflammatory and apoptotic signaling. Y7 also supported epithelial tight-junction integrity in both the eye and colon, consistent with a microbiota-mediated gut–retina axis contribution to protection. Across multiple endpoints, high-dose Y7 matched or exceeded the effects of lutein, positioning probiotics as antioxidant-complementary candidates for retinal protection. These findings highlight *L. reuteri* Y7 as a promising, non-invasive strategy against light-associated retinal injury and warrant confirmation in larger cohorts, mechanistic metabolomics and fecal microbiota transplantation studies, and dose/strain-optimization and safety evaluations to enable translation.

## Figures and Tables

**Table 1:** Systemic antioxidant enzyme activities after 35 days of blue light exposure and interventions.

	N	BL	LU	L	H
Catalase activity (U/mL)	18.48 ± 2.35	2.28 ± 1.63 *	25.40 ± 3.84 ^#^	20.67 ± 1.42 ^#^	24.68 ± 1.96 ^#^
Glutathione peroxidase activity (U/mL)	1.49 ± 0.01	1.15 ± 0.07 *	1.52 ± 0.004 ^#^	1.48 ± 0.03 ^#^	1.58 ± 0.02 ^#^

Data are presented as means ± SEM. *n* = 5 per group. * *p* < 0.05 for BL group compared to the NC group; ^#^ *p* < 0.05 for LU, L, and H groups compared to the BL group. Statistical analysis was performed using one-way ANOVA followed by Tukey’s post hoc test. Group labels: N (normal), BL (blue light), LU (lutein with blue light), L (*L. reuteri* Y7 10^8^ CFU/kg with blue light), H (*L. reuteri* Y7 10^9^ CFU/kg with blue light).

## Data Availability

The original contributions presented in this study are included in the article/Supplementary Material. Further inquiries can be directed to the corresponding author.

## Supplementary Materials

The following supporting information can be downloaded at: https://www.mdpi.com/article/10.3390/antiox14121428/s1, Table S1: Primer sequences used for qPCR.

## Abbreviations

AMD	Age-related macular degeneration
ROS	Reactive oxygen species
CFU	Colony-forming units
ERG	electroretinography
FP	Fundus photography
FFA	Fluorescein angiography
OCT	Optical coherence tomography
RPE	Retinal pigment epithelium
NFL	nerve fiber layer
GCL	ganglion cell layer
IPL	inner plexiform layer
INL	inner nuclear layer
OPL	outer plexiform layer
ONL	outer nuclear layer
IS/OS	inner and outer segments of photoreceptors
GPx	glutathione peroxidase
CAT	catalase
OTUs	operational taxonomic units
PCoA	principal coordinate analysis
LEfSe	linear discriminant analysis effect size
CNV	choroidal neovascularization
*NF-κB p65*	Nuclear Factor kappa-light-chain-enhancer of activated B cells p65 subunit
*MCP-1*	Monocyte Chemoattractant Protein-1
*TNF-α*	Tumor Necrosis Factor-alpha
*IL-6*	Interleukin-6
*IL-1β*	Interleukin-1 beta
*NLRP3*	NOD-, LRR- and pyrin domain-containing protein 3
*IL-18*	Interleukin-18
*Bax*	Bcl-2-associated X protein
*Bcl-2*	B-cell lymphoma 2

## References

[1] Wong W.L., Su X., Li X., Cheung C.M., Klein R., Cheng C.Y., Wong T.Y. (2014). Global prevalence of age-related macular degeneration and disease burden projection for 2020 and 2040: A systematic review and meta-analysis. Lancet Glob. Health.

[2] Ambati J., Fowler B.J. (2012). Mechanisms of age-related macular degeneration. Neuron.

[3] Krigel A., Berdugo M., Picard E., Levy-Boukris R., Jaadane I., Jonet L., Dernigoghossian M., Andrieu-Soler C., Torriglia A., Behar-Cohen F. (2016). Light-induced retinal damage using different light sources, protocols and rat strains reveals LED phototoxicity. Neuroscience.

[4] Nakamura M., Kuse Y., Tsuruma K., Shimazawa M., Hara H. (2017). The Involvement of the Oxidative Stress in Murine Blue LED Light-Induced Retinal Damage Model. Biol. Pharm. Bull..

[5] Marie M., Bigot K., Angebault C., Barrau C., Gondouin P., Pagan D., Fouquet S., Villette T., Sahel J.A., Lenaers G. (2018). Light action spectrum on oxidative stress and mitochondrial damage in A2E-loaded retinal pigment epithelium cells. Cell Death Dis..

[6] Ozkaya E.K., Anderson G., Dhillon B., Bagnaninchi P.O. (2019). Blue-light induced breakdown of barrier function on human retinal epithelial cells is mediated by PKC-zeta over-activation and oxidative stress. Exp. Eye Res..

[7] Morita Y., Miwa Y., Jounai K., Fujiwara D., Kurihara T., Kanauchi O. (2018). Lactobacillus paracasei KW3110 Prevents Blue Light-Induced Inflammation and Degeneration in the Retina. Nutrients.

[8] Westfall S., Lomis N., Kahouli I., Dia S.Y., Singh S.P., Prakash S. (2017). Microbiome, probiotics and neurodegenerative diseases: Deciphering the gut brain axis. Cell Mol. Life Sci..

[9] Morita Y., Jounai K., Sakamoto A., Tomita Y., Sugihara Y., Suzuki H., Ohshio K., Otake M., Fujiwara D., Kanauchi O. (2018). Long-term intake of Lactobacillus paracasei KW3110 prevents age-related chronic inflammation and retinal cell loss in physiologically aged mice. Aging.

[10] Xing Y., Liang S., Zhang L., Ni H., Zhang X., Wang J., Yang L., Song S., Li H.H., Jia C. (2023). Combination of Lactobacillus fermentum NS9 and aronia anthocyanidin extract alleviates sodium iodate-induced retina degeneration. Sci. Rep..

[11] Kammoun S., Rekik M., Dlensi A., Aloulou S., Smaoui W., Sellami S., Trigui K., Gargouri R., Chaari I., Sellami H. (2024). The gut-eye axis: The retinal/ocular degenerative diseases and the emergent therapeutic strategies. Front. Cell Neurosci..

[12] Schiavone N., Isoldi G., Calcagno S., Rovida E., Antiga E., De Almeida C.V., Lulli M. (2025). Exploring the Gut Microbiota-Retina Axis: Implications for Health and Disease. Microorganisms.

[13] Zeng Y., Gao M., Tan X., Wu H., Xiang J., Liu A., Zhang J., Yao Y., Shen T., Zhang T. (2025). The gut-retina axis: Association of dietary index for gut microbiota with diabetic retinopathy in diabetic patients-a cross-sectional study from NHANES 2009-2018. Diabetol. Metab. Syndr..

[14] Nguyen Y., Rudd Zhong Manis J., Ronczkowski N.M., Bui T., Oxenrider A., Jadeja R.N., Thounaojam M.C. (2024). Unveiling the gut-eye axis: How microbial metabolites influence ocular health and disease. Front. Med..

[15] Nair A.B., Jacob S. (2016). A simple practice guide for dose conversion between animals and human. J. Basic. Clin. Pharm..

[16] Yang J., Yang L., Chen R., Zhu Y., Wang S., Hou X., Wei B., Wang Q., Liu Y., Qu J. (2020). A role of color vision in emmetropization in C57BL/6J mice. Sci. Rep..

[17] Klindworth A., Pruesse E., Schweer T., Peplies J., Quast C., Horn M., Glöckner F.O. (2013). Evaluation of general 16S ribosomal RNA gene PCR primers for classical and next-generation sequencing-based diversity studies. Nucleic Acids Res..

[18] Yang D.F., Huang W.C., Wu C.W., Huang C.Y., Yang Y.S.H., Tung Y.T. (2023). Acute sleep deprivation exacerbates systemic inflammation and psychiatry disorders through gut microbiota dysbiosis and disruption of circadian rhythms. Microbiol. Res..

[19] Ferguson L.R., Grover S., Dominguez J.M., Balaiya S., Chalam K.V. (2014). Retinal thickness measurement obtained with spectral domain optical coherence tomography assisted optical biopsy accurately correlates with ex vivo histology. PLoS ONE.

[20] Cheng J., Sohn E.H., Jiao C., Adler K.L., Kaalberg E.E., Russell S.R., Mullins R.F., Stone E.M., Tucker B.A., Han I.C. (2018). Correlation of Optical Coherence Tomography and Retinal Histology in Normal and Pro23His Retinal Degeneration Pig. Transl. Vis. Sci. Technol..

[21] Miller J.W., Bagheri S., Vavvas D.G. (2017). Advances in Age-related Macular Degeneration Understanding and Therapy. US Ophthalmic Rev..

[22] Nwanna E.E., Ibukun E.O., Oboh G. (2019). Nutritional content of selected species of tropical eggplant fruit (Solanum spp) diet Attenuates hepatic inflammation in high-fat fed male Wistar rats induced with streptozotocin. Food Sci. Nutr..

[23] Organisciak D.T., Vaughan D.K. (2010). Retinal light damage: Mechanisms and protection. Prog. Retin. Eye Res..

[24] Nakamura M., Yako T., Kuse Y., Inoue Y., Nishinaka A., Nakamura S., Shimazawa M., Hara H. (2018). Exposure to excessive blue LED light damages retinal pigment epithelium and photoreceptors of pigmented mice. Exp. Eye Res..

[25] Imamura Y., Noda S., Hashizume K., Shinoda K., Yamaguchi M., Uchiyama S., Shimizu T., Mizushima Y., Shirasawa T., Tsubota K. (2006). Drusen, choroidal neovascularization, and retinal pigment epithelium dysfunction in SOD1-deficient mice: A model of age-related macular degeneration. Proc. Natl. Acad. Sci. USA.

[26] Bakri S.J., Bektas M., Sharp D., Luo R., Sarda S.P., Khan S. (2023). Geographic atrophy: Mechanism of disease, pathophysiology, and role of the complement system. J. Manag. Care Spec. Pharm..

[27] Rajanala K., Dotiwala F., Upadhyay A. (2023). Geographic atrophy: Pathophysiology and current therapeutic strategies. Front. Ophthalmol..

[28] Shang Y.M., Wang G.S., Sliney D., Yang C.H., Lee L.L. (2014). White light-emitting diodes (LEDs) at domestic lighting levels and retinal injury in a rat model. Environ. Health Perspect..

[29] Lin C.H., Wu M.R., Li C.H., Cheng H.W., Huang S.H., Tsai C.H., Lin F.L., Ho J.D., Kang J.J., Hsiao G. (2017). Editor’s Highlight: Periodic Exposure to Smartphone-Mimic Low-Luminance Blue Light Induces Retina Damage Through Bcl-2/BAX-Dependent Apoptosis. Toxicol. Sci..

[30] Walton S.P., Wu M., Gredell J.A., Chan C. (2010). Designing highly active siRNAs for therapeutic applications. FEBS J..

[31] Robson A.G., El-Amir A., Bailey C., Egan C.A., Fitzke F.W., Webster A.R., Bird A.C., Holder G.E. (2003). Pattern ERG correlates of abnormal fundus autofluorescence in patients with retinitis pigmentosa and normal visual acuity. Invest. Ophthalmol. Vis. Sci..

[32] Roberts J.E., Dennison J. (2015). The Photobiology of Lutein and Zeaxanthin in the Eye. J. Ophthalmol..

[33] Mrowicka M., Mrowicki J., Kucharska E., Majsterek I. (2022). Lutein and Zeaxanthin and Their Roles in Age-Related Macular Degeneration-Neurodegenerative Disease. Nutrients.

[34] Age-Related Eye Disease Study 2 Research Group (2013). Lutein + zeaxanthin and omega-3 fatty acids for age-related macular degeneration: The Age-Related Eye Disease Study 2 (AREDS2) randomized clinical trial. JAMA.

[35] Chew E.Y., Clemons T.E., Agron E., Domalpally A., Keenan T.D.L., Vitale S., Weber C., Smith D.C., Christen W., Group A.R. (2022). Long-term Outcomes of Adding Lutein/Zeaxanthin and omega-3 Fatty Acids to the AREDS Supplements on Age-Related Macular Degeneration Progression: AREDS2 Report 28. JAMA Ophthalmol..

[36] Chan Y.J., Hsiao G., Wan W.N., Yang T.M., Tsai C.H., Kang J.J., Lee Y.C., Fang T.C., Cheng Y.W., Li C.H. (2023). Blue light exposure collapses the inner blood-retinal barrier by accelerating endothelial CLDN5 degradation through the disturbance of GNAZ and the activation of ADAM17. Fluids Barriers CNS.

[37] Lin C.F., Hsu Y.A., Chou Y.L., Chen Y.C., Lin E.S., Tien P.T., Chen J.J., Wu M.Y., Lin C.H., Lin H.J. (2025). Harnessing Lactiplantibacillus plantarum EP21 and its membrane vesicles to inhibit myopia development. Gut Microbes.

[38] Prisciandaro L.D., Geier M.S., Chua A.E., Butler R.N., Cummins A.G., Sander G.R., Howarth G.S. (2012). Probiotic factors partially prevent changes to caspases 3 and 7 activation and transepithelial electrical resistance in a model of 5-fluorouracil-induced epithelial cell damage. Support. Care Cancer.

[39] Lin C.H., Wu M.R., Huang W.J., Chow D.S., Hsiao G., Cheng Y.W. (2019). Low-Luminance Blue Light-Enhanced Phototoxicity in A2E-Laden RPE Cell Cultures and Rats. Int. J. Mol. Sci..

[40] Anderson R.C., Cookson A.L., McNabb W.C., Park Z., McCann M.J., Kelly W.J., Roy N.C. (2010). Lactobacillus plantarum MB452 enhances the function of the intestinal barrier by increasing the expression levels of genes involved in tight junction formation. BMC Microbiol..

[41] Zhao Z.C., Zhou Y., Tan G., Li J. (2018). Research progress about the effect and prevention of blue light on eyes. Int. J. Ophthalmol..

[42] Chakravarthy H., Georgyev V., Wagen C., Hosseini A., Matsubara J. (2024). Blue light-induced phototoxicity in retinal cells: Implications in age-related macular degeneration. Front. Aging Neurosci..

[43] Zhang Y., Zhu X., Yu X., Novak P., Gui Q., Yin K. (2023). Enhancing intestinal barrier efficiency: A novel metabolic diseases therapy. Front. Nutr..

[44] Gou H.Z., Zhang Y.L., Ren L.F., Li Z.J., Zhang L. (2022). How do intestinal probiotics restore the intestinal barrier?. Front. Microbiol..

[45] Zhang H., Mo Y. (2023). The gut-retina axis: A new perspective in the prevention and treatment of diabetic retinopathy. Front. Endocrinol..

[46] Scuderi G., Troiani E., Minnella A.M. (2021). Gut Microbiome in Retina Health: The Crucial Role of the Gut-Retina Axis. Front. Microbiol..

[47] Dalile B., Van Oudenhove L., Vervliet B., Verbeke K. (2019). The role of short-chain fatty acids in microbiota-gut-brain communication. Nat. Rev. Gastroenterol. Hepatol..

[48] Chen N., Wu J., Wang J., Piri N., Chen F., Xiao T., Zhao Y., Sun D., Kaplan H.J., Shao H. (2021). Short chain fatty acids inhibit endotoxin-induced uveitis and inflammatory responses of retinal astrocytes. Exp. Eye Res..

[49] Louis P., Flint H.J. (2017). Formation of propionate and butyrate by the human colonic microbiota. Environ. Microbiol..

[50] Zhang Y., Wang T., Wan Z., Bai J., Xue Y., Dai R., Wang M., Peng Q. (2023). Alterations of the intestinal microbiota in age-related macular degeneration. Front. Microbiol..

[51] Luo W., Skondra D. (2023). Elucidating the Role of the Microbiome in Ocular Diseases. Am. J. Pathol..

[52] Qin X., Sun J., Chen S., Xu Y., Lu L., Lu M., Li J., Ma Y., Lou F., Zou H. (2024). Gut microbiota predict retinopathy in patients with diabetes: A longitudinal cohort study. Appl. Microbiol. Biotechnol..

[53] Gong H., Zuo H., Wu K., Gao X., Lan Y., Zhao L. (2025). Systemic and Retinal Protective Effects of Butyrate in Early Type 2 Diabetes via Gut Microbiota-Lipid Metabolism Interaction. Nutrients.

[54] Zinkernagel M.S., Zysset-Burri D.C., Keller I., Berger L.E., Leichtle A.B., Largiader C.R., Fiedler G.M., Wolf S. (2017). Association of the Intestinal Microbiome with the Development of Neovascular Age-Related Macular Degeneration. Sci. Rep..

[55] Ciurariu E., Tirziu A.T., Varga N.I., Hirtie B., Alexandru A., Ivan C.S., Nicolescu L. (2025). Short-Chain Fatty Acids and the Gut-Retina Connection: A Systematic Review. Int. J. Mol. Sci..

[56] Ndongo S., Tall M.L., Ngom I.I., Delerce J., Levasseur A., Raoult D., Fournier P.E., Khelaifia S. (2019). Olsenella timonensis sp. nov., a new bacteria species isolated from the human gut microbiota. New Microbes New Infect..

[57] Parker A., Romano S., Ansorge R., Aboelnour A., Le Gall G., Savva G.M., Pontifex M.G., Telatin A., Baker D., Jones E. (2022). Fecal microbiota transfer between young and aged mice reverses hallmarks of the aging gut, eye, and brain. Microbiome.

[58] Di Vincenzo F., Del Gaudio A., Petito V., Lopetuso L.R., Scaldaferri F. (2024). Gut microbiota, intestinal permeability, and systemic inflammation: A narrative review. Intern. Emerg. Med..

[59] Larsen J.M. (2017). The immune response to Prevotella bacteria in chronic inflammatory disease. Immunology.

[60] Sun L., Jia H., Li J., Yu M., Yang Y., Tian D., Zhang H., Zou Z. (2019). Cecal Gut Microbiota and Metabolites Might Contribute to the Severity of Acute Myocardial Ischemia by Impacting the Intestinal Permeability, Oxidative Stress, and Energy Metabolism. Front. Microbiol..

[61] O’Callaghan A., van Sinderen D. (2016). Bifidobacteria and Their Role as Members of the Human Gut Microbiota. Front. Microbiol..

[62] Koh A., De Vadder F., Kovatcheva-Datchary P., Backhed F. (2016). From Dietary Fiber to Host Physiology: Short-Chain Fatty Acids as Key Bacterial Metabolites. Cell.

[63] Li T., Chiang J.Y. (2015). Bile acids as metabolic regulators. Curr. Opin. Gastroenterol..

[64] Song Z., Cai Y., Lao X., Wang X., Lin X., Cui Y., Kalavagunta P.K., Liao J., Jin L., Shang J. (2019). Taxonomic profiling and populational patterns of bacterial bile salt hydrolase (BSH) genes based on worldwide human gut microbiome. Microbiome.

[65] Pols T.W., Nomura M., Harach T., Lo Sasso G., Oosterveer M.H., Thomas C., Rizzo G., Gioiello A., Adorini L., Pellicciari R. (2011). TGR5 activation inhibits atherosclerosis by reducing macrophage inflammation and lipid loading. Cell Metab..

[66] Wang K., Liao M., Zhou N., Bao L., Ma K., Zheng Z., Wang Y., Liu C., Wang W., Wang J. (2019). Parabacteroides distasonis Alleviates Obesity and Metabolic Dysfunctions via Production of Succinate and Secondary Bile Acids. Cell Rep..

[67] Dodd D., Spitzer M.H., Van Treuren W., Merrill B.D., Hryckowian A.J., Higginbottom S.K., Le A., Cowan T.M., Nolan G.P., Fischbach M.A. (2017). A gut bacterial pathway metabolizes aromatic amino acids into nine circulating metabolites. Nature.

[68] Roager H.M., Licht T.R. (2018). Microbial tryptophan catabolites in health and disease. Nat. Commun..

[69] Yano J.M., Yu K., Donaldson G.P., Shastri G.G., Ann P., Ma L., Nagler C.R., Ismagilov R.F., Mazmanian S.K., Hsiao E.Y. (2015). Indigenous bacteria from the gut microbiota regulate host serotonin biosynthesis. Cell.

[70] Kim C.C., Lunken G.R., Kelly W.J., Patchett M.L., Jordens Z., Tannock G.W., Sims I.M., Bell T.J., Hedderley D., Henrissat B. (2019). Genomic insights from Monoglobus pectinilyticus: A pectin-degrading specialist bacterium in the human colon. ISME J..

[71] Kim C.C., Kelly W.J., Patchett M.L., Tannock G.W., Jordens Z., Stoklosinski H.M., Taylor J.W., Sims I.M., Bell T.J., Rosendale D.I. (2017). Monoglobus pectinilyticus gen. nov., sp. nov., a pectinolytic bacterium isolated from human faeces. Int. J. Syst. Evol. Microbiol..

[72] Rios-Covian D., Ruas-Madiedo P., Margolles A., Gueimonde M., de Los Reyes-Gavilan C.G., Salazar N. (2016). Intestinal Short Chain Fatty Acids and their Link with Diet and Human Health. Front. Microbiol..

[73] Zhao T., Zhao P., West J.W., Bernard J.K., Cross H.G., Doyle M.P. (2006). Inactivation of enterohemorrhagic Escherichia coli in rumen content- or feces-contaminated drinking water for cattle. Appl. Environ. Microbiol..

[74] Mathias B., Gehring W.J., Palmer C. (2017). Auditory N1 reveals planning and monitoring processes during music performance. Psychophysiology.

[75] Lin J.B., Kubota S., Mostoslavsky R., Apte R.S. (2018). Role of Sirtuins in Retinal Function Under Basal Conditions. Adv. Exp. Med. Biol..

[76] Miranda A.S., Cordeiro T.M., Dos Santos Lacerda Soares T.M., Ferreira R.N., Simoes E.S.A.C. (2017). Kidney-brain axis inflammatory cross-talk: From bench to bedside. Clin. Sci..

[77] Everts B., Pearce E.J. (2014). Metabolic control of dendritic cell activation and function: Recent advances and clinical implications. Front. Immunol..

[78] Louis P., Hold G.L., Flint H.J. (2014). The gut microbiota, bacterial metabolites and colorectal cancer. Nat. Rev. Microbiol..

[79] Everard A., Belzer C., Geurts L., Ouwerkerk J.P., Druart C., Bindels L.B., Guiot Y., Derrien M., Muccioli G.G., Delzenne N.M. (2013). Cross-talk between Akkermansia muciniphila and intestinal epithelium controls diet-induced obesity. Proc. Natl. Acad. Sci. USA.

[80] Derrien M., Vaughan E.E., Plugge C.M., de Vos W.M. (2004). Akkermansia muciniphila gen. nov., sp. nov., a human intestinal mucin-degrading bacterium. Int. J. Syst. Evol. Microbiol..

[81] Severns P.M., Mundt C.C. (2022). Delays in Epidemic Outbreak Control Cost Disproportionately Large Treatment Footprints to Offset. Pathogens.

[82] Plovier H., Everard A., Druart C., Depommier C., Van Hul M., Geurts L., Chilloux J., Ottman N., Duparc T., Lichtenstein L. (2017). A purified membrane protein from Akkermansia muciniphila or the pasteurized bacterium improves metabolism in obese and diabetic mice. Nat. Med..

[83] Vastenhouw N.L., Cao W.X., Lipshitz H.D. (2019). The maternal-to-zygotic transition revisited. Development.

[84] Rovira A., Auger C. (2022). Routine Gadolinium Use for MRI Follow-Up of Multiple Sclerosis: Counterpoint-Gadolinium Should Not Always Be Used to Assess Disease Activity. AJR Am. J. Roentgenol..

[85] Hauer J., Fischer U., Auer F., Borkhardt A. (2020). Regional BCG vaccination policy in former East- and West Germany may impact on both severity of SARS-CoV-2 and incidence of childhood leukemia. Leukemia.

[86] Martens E.C., Koropatkin N.M., Smith T.J., Gordon J.I. (2009). Complex glycan catabolism by the human gut microbiota: The Bacteroidetes Sus-like paradigm. J. Biol. Chem..

[87] Wexler H.M. (2007). Bacteroides: The good, the bad, and the nitty-gritty. Clin. Microbiol. Rev..

[88] Loutfi D., Andersson N., Law S., Salsberg J., Haggerty J., Kgakole L., Cockcroft A. (2019). Can social network analysis help to include marginalised young women in structural support programmes in Botswana? A mixed methods study. Int. J. Equity Health.

[89] Round J.L., Mazmanian S.K. (2009). The gut microbiota shapes intestinal immune responses during health and disease. Nat. Rev. Immunol..

