# Supplementary material for: Probiotic Lactobacillus reuteri Y7 Protects Against Blue Light–Induced Retinal Degeneration via Antioxidant Defense, Anti-Inflammatory Action, and Gut–Retina Axis Modulation
Source: Antioxidants (Basel). 2025 Nov 27;14(12):1428. doi: 10.3390/antiox14121428 (PMC12729536; doi:10.3390/antiox14121428)
Supplement: Supplementary file 1 [file antioxidants-14-01428-s001.zip › antioxidants-3880765-supplementary.pdf]

Table S1. Primer sequences used for qPCR.

Gene	Accession No.	Forward primer (5’-3’)	Reverse primer (5’-3’)
NF-kB p65	NM_009045	TCCTGTICGAGTCTCCATGCAG	GGTCTCATAGGTCCTTITGCGC
MCP-1	NM 011333	GCTACAAGAGGATCACCAGCAG	GTCTGGACCCATTCCTICTTGG
TNF-a	NM 001278601.1	GGTGCCTATGTCTCAGCCTCTT	GCCATAGAACTGATGAGAGGGAG
IL-6	NM 031168	TACCACTTCACAAGIOGGAGG	CTGCAAGTGCATCATCGTIGTTC
IL-1B	NM 008361.4	TGCCACCTTITGACAGTGATG	AAGGTCCACGGGAAAGACAC
NLRP3	NM 145827	TCACAACTCGCCCAAGGAGGAA	AAGAGACCACGGCAGAAGCTAG
Caspase1	NM 009807.2	GGGCAAAGAGGAAGCAATITATC	GTGCCTTGTCCATAGCAGTAA
IL-18	NM 008360	GACAGCCTGTGTTCGAGGATATG	TGTTCTTACAGGAGAGGGTAGAC
Bax	NM 007527.4	TGCAGAGGATGATTGCTGAC	CAAAGTAGAAGAGGGCAACCA
Bcl-2	NM 009741.5	AGAGCGTCAACAGGGAGAT	GGGCCATATAGTTCCACAAAGG
Caspase3	XM 017312543.3	GGAGTCTGACTGGAAAGCCGAA	CTTCTGGCAAGCCATCTCCTCA
Occludin	NM 001404038.1	CTCCCATCCGAGTTICAGGT	GCTGTCGCCTAAGGAAAGAG
Claudin-1	NM 016674.4	GTTTGCAGAGACCCCATCAC	AGAAGCCAGGATGAAACCCA
ZO-1	NM 001417372.1	GCACCATGCCTAAAGCTGTC	ACTCAACACACCACCATTGC
GAPDH	NM 001411843.1	GGAGCCAAACOGGTCATCATCTC	GAGGGGCCATCCACAGTCTTCT
